# Measure what matters: considerations for outcome measurement of care coordination for children with neurodevelopmental disabilities and medical complexity

**DOI:** 10.3389/fpubh.2023.1280981

**Published:** 2023-11-03

**Authors:** Dércia Materula, Genevieve Currie, Xiao Yang Jia, Brittany Finlay, Catherine Richard, Meridith Yohemas, Gina Lachuk, Myka Estes, Tammie Dewan, Sarah MacEachern, Nadine Gall, Ben Gibbard, Jennifer D. Zwicker

**Affiliations:** ^1^School of Public Policy, University of Calgary, Calgary, AB, Canada; ^2^School of Nursing and Midwifery, Mount Royal University, Calgary, AB, Canada; ^3^Alberta Health Services, Calgary, AB, Canada; ^4^Department of Pediatrics, Cumming School of Medicine, University of Calgary, Calgary, AB, Canada; ^5^Cumming School of Medicine, University of Calgary, Calgary, AB, Canada; ^6^Faculty of Kinesiology, University of Calgary, Calgary, AB, Canada

**Keywords:** neurodevelopmental disorders, medical complexity, children, caregivers, care coordination, quality of life, resource utilization

## Abstract

**Introduction:**

Care Coordination (CC) is a significant intervention to enhance family’s capacity in caring for children with neurodevelopmental disability and medical complexity (NDD-MC). CC assists with integration of medical and behavioral care and services, partnerships with medical and community-based supports, and access to medical, behavioral, and educational supports and services. Although there is some consensus on the principles that characterize optimal CC for children with NDD-MC, challenges remain in measuring and quantifying the impacts of CC related to these principles. Two key challenges include: (1) identification of measures that capture CC impacts from the medical system, care provider, and family perspectives; and (2) recognition of the important community context outside of a hospital or clinical setting.

**Methods:**

This study used a multilevel model variant of the triangulation mixed methods design to assess the impact of a CC project implemented in Alberta, Canada, on family quality of life, resource use, and care integration at the broader environmental and household levels. At the broader environmental level, we used linked administrative data. At the household level we used quantitative pre-post survey datasets, and aggregate findings from qualitative interviews to measure group-level impacts and an embedded multiple-case design to draw comparisons, capture the nuances of children with NDD-MC and their families, and expand on factors driving the high variability in outcome measures. Three theoretical propositions formed the basis of the analytical strategy for our case study evidence to explore factors affecting the high variability in outcome measures.

**Discussion:**

This study expanded on the factors used to measure the outcomes of CC and adds to our understanding of how CC as an intervention impacts resource use, quality of life, and care integration of children with NDD-MC and their families. Given the heterogeneous nature of this population, evaluation studies that account for the variable and multi-level impacts of CC interventions are critical to inform practice, implementation, and policy of CC for children with NDD-MC.

## Introduction

Children with medical complexity (CMC) are a subset of children and youth with special health care needs (CSHCN). Due to the severity of their health care condition, which requires care above the levels for typically developing children, CMC are a priority population for healthcare policy (1). The definitions of CMC often meet four criteria: (i) severe functional limitations, (ii) severe chronic health conditions, commonly linked to medical fragility; (iii) high care needs placing high burden on families, and (iv) high resource use requiring support from multiple sectors (2–5). Some CMC, have neurodevelopmental disability [NDD-MC (1)]. Children with NDD-MC have functional needs spanning physical, learning, social, behavioral, and emotional domains and require supports and services to reduce barriers and limitations in their ability to participate fully within society. In Canada, provincial governments provide the majority of health, social, and education services important for meeting the functional needs of NDD-MC (6). Unfortunately, this system has been long characterized as complex, fragmented, and challenging to navigate (6). The United Nations Special Rapporteur on the Rights of Persons with Disabilities recognized the fragmented delivery of supports and services (6). In 2019, they urgently recommended governments coordinate efforts to effectively safeguard the rights of persons with disabilities. Furthermore, system fragilities in addressing CMC’s needs gained greater prominence after the coronavirus pandemic, as NDD-MC were disproportionately impacted (7, 8), the gaps in systems of care became more salient and the adequacy of financial supports was in question (6). As such, care coordination (CC) for CMC becomes increasingly important due to its role in addressing system fragmentation (9).

While importance of CC is increasing, there is currently a lack of understanding of CC outcome measurement. The imperative to have a better understanding of CC outcome measurement arises due to several factors. Clinicians and health care researchers have struggled to consistently define and measure outcomes from CMC CC interventions (10, 11). Definitions are context-specific, often leading to variable thresholds in eligibility criteria for support services (5). Given the lack of consensus on defining CMC and measuring outcomes at the population and individual level, various tools, including diagnosis classification schemes and questionnaires, are used to identify CMC (12). The lack of uniformity in CMC definitions and outcome measurement presents challenges in evaluation research limiting scalability and replicability of CC interventions (5). Furthermore, despite some level of consensus on the impact of CC in addressing system fragmentation (9), evaluating the effectiveness of CC interventions remains challenging for researchers and clinical practitioners. The plurality of implementation models, inconsistencies in definitions, and often limited availability of adequate outcome measures present difficulties in CC evaluation efforts. Since outcome measures and CMC-related definitions are context-specific, findings from studies evaluating the impact of CC interventions may vary (13, 14). This underscores the need for researchers and clinical professionals to improve their understanding of the contexts in which they operate to ensure the integration of appropriate outcome measures in evaluation research. This study focuses on addressing some of the challenges related to the outcome measurement and evaluation of CC interventions for NDD-MC.

Several key frameworks guide our analysis of NDD-MC outcome mefasurement for CC interventions. An implementation model of CC, including its functions and characteristics was instrumental for our research study in two ways (12). First, we adopted Antonelli et al.’s definition of CC, which is understood to be: “patient and family-centered, assessment-driven, team-based activity designed to meet the needs of children and youth while enhancing the caregiving capabilities of families. Care coordination addresses interrelated medical, social, developmental, behavioral, educational and financial needs to achieve optimal health and wellness outcomes” (12, p. 8). Second, we used the Antonelli et al.’s Outcomes and Needed Measures multidisciplinary framework (12), to guide data analysis towards the evaluation of the NDD-CC project. This framework recognized the multidisciplinary nature of CC and the various environmental processes, structures, and outcomes involved in providing CC to families with CMC (12).

**Figure 1:**
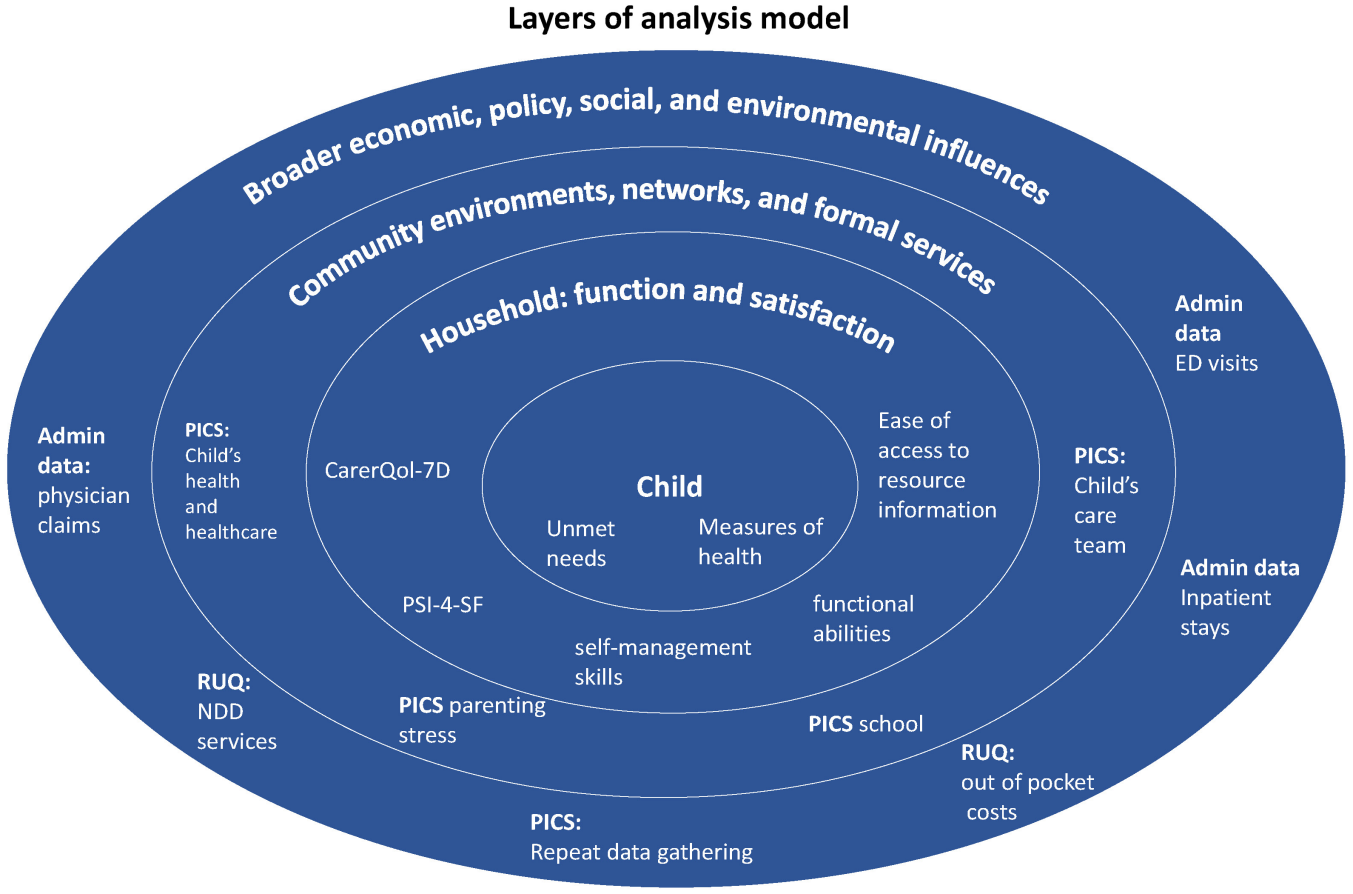
This model provides an overview of the different levels of analysis of our study. At each level, the measures used in the data analysis were identified. Adaptation of the Center for Community Child Health’s (Platforms) Ecological model.

Additionally, recognition of the multilevel impacts that occur was a critical lens to incorporate in CC outcome measurement. The Center for Community Child Health’s (Platforms) Ecological model also guides our data collection and analysis (14). This model is an adaptation of Bronfenbrenner’s (14–16) ecological systems theory. This theory looks at a child’s development within the context of the system of relationships that form his or her environment. Bronfenbrenner’s theory defines complex “layers” of environment, each having an effect on a child’s development: the interaction between factors in the child’s maturing biology, their immediate family/community environment, and the societal landscape (15). Changes or conflict in any one layer will ripple throughout other layers (17). Our analysis focuses on three levels: (i) Broader economic, policy, social, and environmental influences; (ii) Community environments, networks, and formal services; and, (iii) household: function and satisfaction (Figure 1). We take a multidisciplinary approach to measuring CC by considering external influences that affect the CC interventions. As such, there is an intersection of the multidisciplinary nature of the two frameworks described.

The rationale for the inclusion of this framework as the basis for our analysis is rooted in the similarity to the ecological model. Utilizing these frameworks, this study contributes to expanding the body of knowledge on NDD-MC outcome measurement for CC interventions. We focus our analysis on the evaluation of the Neurodevelopmental Disorders Care Coordination (NDD-CC) Project implemented in a Western Canadian province (Alberta).

## Materials and methods

### Study design

**Figure 2:**
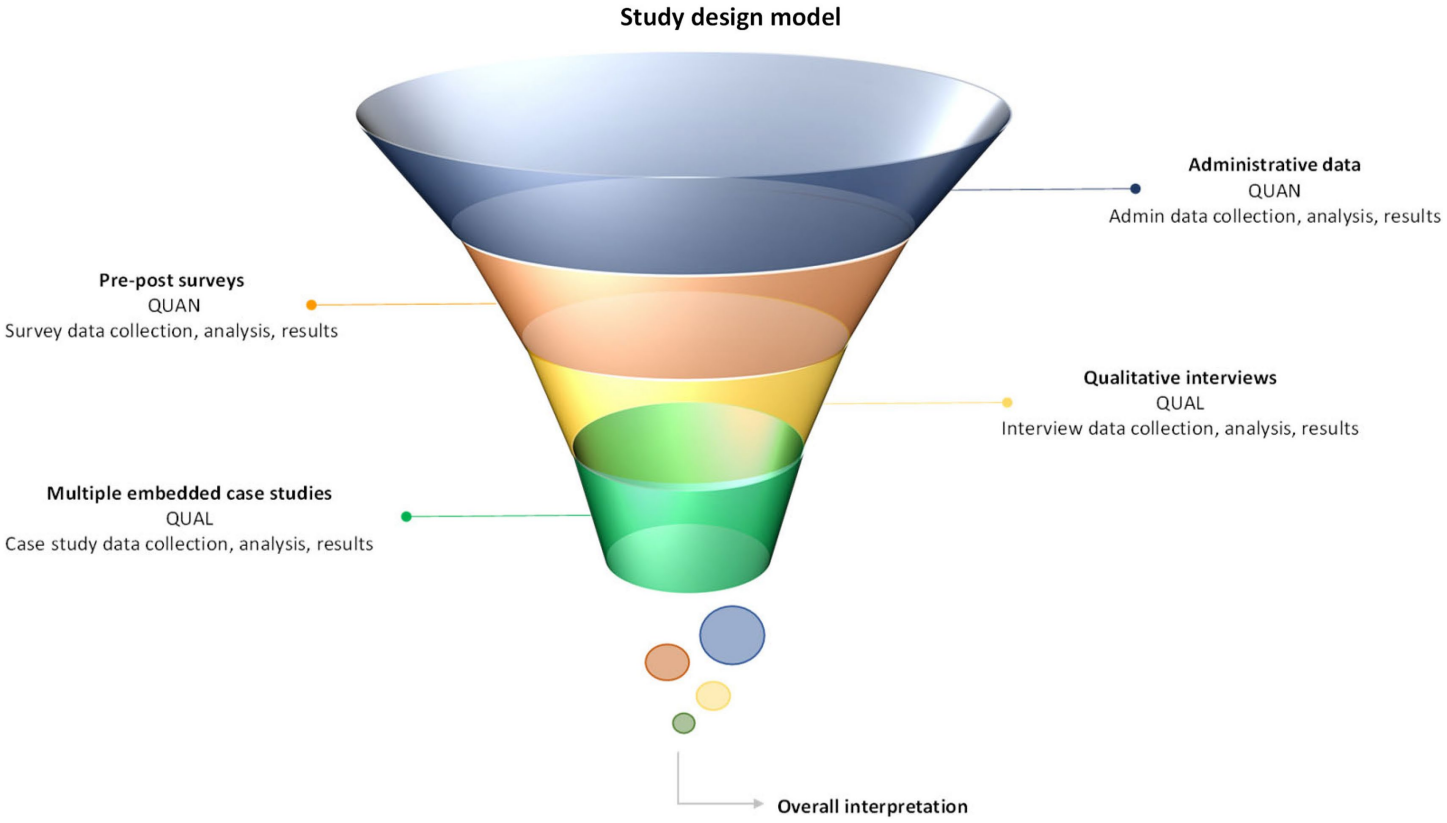
Multilevel model variant of the triangulation mixed methods study design model.

This study used the multilevel model variant of the triangulation mixed methods design (Figure 2) (18) exploring appropriate measurement domains that describe how CC as an intervention impacts children with NDD-MC and their families. Our research question was: What domains of measurement are important for describing the impact of a CC intervention at a system and household level? Two secondary research questions were defined to assist in answering the overall research question: (1) What impacts does the NDD-CC have on health service utilization? (2) What domains of measurement are important to describe the impact of NDD-CC for families?

This project received ethics approval through the University of Calgary CHREB (REB18-0743) and AHS Data Disclosure Agreement & Administrative Approval. Informed consent was obtained from all caregivers enrolled in the study to collect and use their data.

### Care coordination measurement evaluation frameworks

To assess the impact of NDD-CC, the study data collection and analysis was guided by the Center for Community Child Health’s (Platforms) Ecological model where at the system and household level data was collected on resource use, care integration, and quality of life. Building off an established measurement framework, we adapted the Measuring Care Coordination: Outcomes and Needed Measures Framework (12) to guide the evaluation of the impact of the NDD-CC intervention. This framework combines a family-centered and health systems approach to assess CC interventions across four dimensions: satisfaction, function, clinical, and costs of care (12). An adapted framework was created maintaining the dimensions of value and outcome measures that the research team had the capacity to report on. Relevant questions from the different survey measures and the administrative data linkage were embedded into this adaptation. All dimensions that required information that we did not possess, including achieve patient/family goals, increase provider and staff satisfaction, support achievement of optimal developmental trajectory, increase activity: developmental screening and health promotion (Early and Periodic Screening, Diagnosis, and Treatment, and Reduce duplication of tests, services), were excluded. The framework presents baseline and 12-month data for each dimension of value and outcome measure to track changes.

### Theoretical propositions for multi method study

We defined theoretical propositions based on the frameworks to evaluate the multi-methods data collected.

#### Broader economic, policy, social, and environmental influences

Theoretical proposition: Equipping caregivers with resource information specific to their children’s NDDs enables families’ to access appropriate resources and improves management of chronic health condition (12).

#### Community environments, networks and formal services

Theoretical proposition: The quality of care integration experienced by families with children with NDD-MC is determined by the degree of family engagement with care teams in care planning for their children with NDD-MC (12).

#### Household: function and satisfaction

Theoretical proposition: To improve family quality of life, CC interventions should be flexible to address the changeability of children with NDD-MC’s medical, educational, and social care needs (12).

### Clinical setting eligibility and recruitment

**Figure 3:**
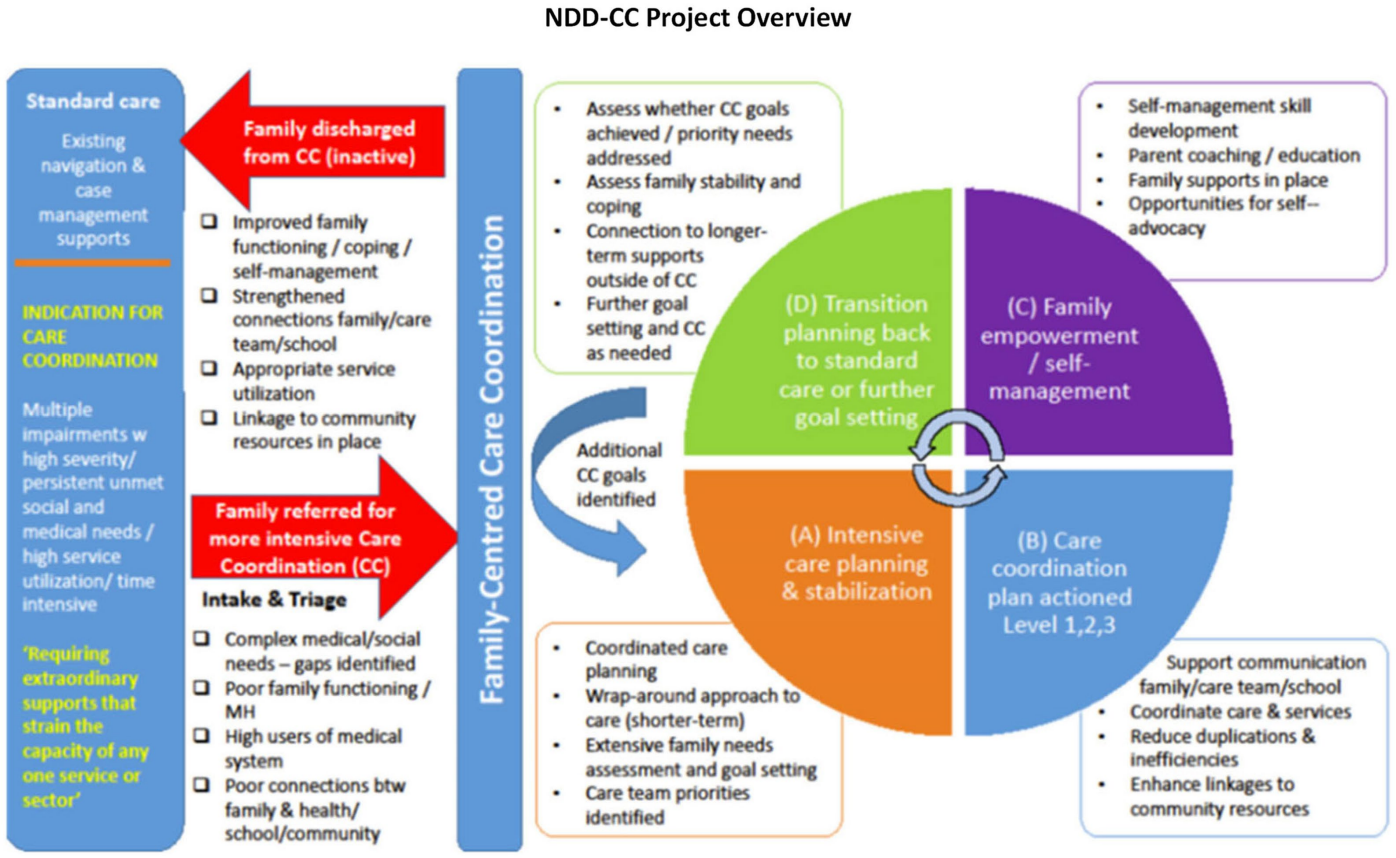
NDD-CC project overview adapted from an established model of care coordination designed by Boston Children’s Hospital (BCH), rigorously tested and researched among a similar patient population in Boston. We are specifically focusing on the evaluation of this program.

This study assessed the impact of the NDD-CC project on children with NDD-MC and their families. We recruited families enrolled in the NDD-CC intervention implemented at the Alberta Children’s Hospital in Alberta, Canada (19). The 12-month intervention (Figure 3) supports families with children with NDD-MC with co-occurring ADHD and/or ASD in navigating the continuum of care across health, education, disability, social, and community service settings (20).

Recruitment and eligibility are described in detail in Gall et al. (19). Briefly, care coordinators reviewed referrals from families with children with NDD-MC provided by community or subspecialist pediatricians. Inclusion criteria included: children aged 0–17 years with an ASD and/or ADHD diagnosis and concurrent medical complexity, residing in the Southern Alberta catchment with high resource use and unmet needs across health, education, and social sectors. Once enrolled in the NDD-CC project, caregivers were invited to participate in this evaluation study. Care coordinators shared the contact details of caregivers interested in the study with the research team who obtained informed consent from all caregivers before data collection took place.

### Data sources

This study relied on information from linked administrative datasets, pre-post surveys, and qualitative semi-structured interviews to construct the case studies. Integrating various sources of evidence allowed the research team to establish construct validity (21). The case studies, formatted as vignettes, focused on the following domains: resource use, quality of life, and care integration. Key informants (including medical doctors and nurses) with knowledge of and experience in managing and implementing care interventions for children with NDD-MC were consulted and reviewed the case studies to further enhance construct validity (21).

#### Administrative data

Data was obtained from linked Alberta health administrative databases through Alberta Health Services (AHS). The linked data were used to assess the desired outcomes before and after CC, which included Emergency Department visits, hospitalizations, hospital length of stay, and caregivers’ workdays lost (22). Outcomes were analyzed for each child for the period of 1 year before and after the baseline interview (proxy for pre- and post-CC). Physician costs were also estimated through amount paid in the physician claims data, which recorded dates of claims, billed fee for service codes, and type of provider setting. Missing cost data was imputed based on fees in the Alberta Medical Association guide (22) for the associated billing codes, applying conservative estimates where applicable. Twelve-month physician claims costs were totaled for each child, pre- and post-CC. The number of unique claims’ dates was used as a proxy for the number of days families attended appointments, which could be a proxy indicator of time off requirements for caregiving.

The inpatient costs were estimated by multiplying the Alberta cost per weighted case (CPWC) for the corresponding fiscal year by the resource intensity weight (RIW) value assigned to each inpatient case based on the Canadian Institute for Health Information (CIHI) grouping methodology (23) (Table 1). The RIW value estimates the amount of hospital resources consumed by a given patient relative to that of an average inpatient case (RIW = 1.0) (23). The CPWC covers direct and indirect hospital costs (i.e., administration, staff, supplies, technology, and equipment) but does not include physician costs (23). Costs were adjusted for inflation to 2022 Canadian dollars based on the Statistics Canada consumer price index for health and personal goods (24) (Table 2). The total inpatient costs of the aggregate sample 1 year before and after CC were determined. We also looked at the Case Mix Group (CMG) classification, which groups inpatient stays with comparable clinical and resource use characteristics (25).

**Table 1:** Cost per weighted case of Alberta acute hospitalizations (60).

**Fiscal Year**	**CPWC**
2017–2018	$8,167
2018–2019	$8,271
2019–2020	$8,114
2020–2021	$9,284
2021–2022	$9,220

**Table 2:** Alberta consumer price index for health and personal goods (*Consumer Price Index, Annual Average, Not Seasonally Adjusted*, 2023).

**Year**	**CPI**
2017	134.1
2018	136.5
2019	138.1
2020	139.9
2021	141.1
2022	144.5

#### Survey data

Pre- and post- interviewer-administered surveys described the children’s quality of life, resource use, and care integration experiences. The completion of all questionnaires was not compulsory; caregivers were provided the option to skip the surveys if they did not wish to complete them. The Research Electronic Data Capture (REDCap) (26, 27) tool hosted at the University of Calgary was used to collect and store survey data. Respondents were assigned a unique identifier to maintain patient confidentiality. Where applicable, all validated measurements were analyzed adhering to scoring guidelines provided by the different developers. Measures included quality of life measures for the child [The Euroqol-5-Dimensions Youth (EQ-5D-Y) including the visual analogue scale EQ-VAS (28)], quality of life for the caregiver [Care-related Quality of Life -7D (29–31)], caregiver stress [The Parenting Stress Index 4th Edition Short Form (PSI-4-SF) (32, 33)], care integration [Pediatric Integrated Care Survey (PICS) (34)], and resource use [The Resource Use Questionnaire (RUQ) (35)]. All survey questionnaires were analyzed in accordance with the guidelines provided by the developers (28–30, 32, 34, 35).

### Qualitative data

#### Semi-structured interviews

Qualitative descriptions using semi-structured interviews were used to describe the experience of caregivers with children with NDD-MC (36). Qualitative data provided contextual information for 19 caregivers who completed the semi-structured interviews from August 2020 to January 2021. Eligibility for the interviews required family’s active participation of at least 4 months in the NDD-CC project. Maximum variation sampling focused on select demographic information such as age and number of children with medical complexities, type of NDD-MC, number of caregivers, marital status, income level, and rural or urban dwelling guided recruitment of participants. Phenomena were described from caregivers as well as their interactions with contextual factors as part of qualitative description (37). Caregivers described their experiences of resource use (lack of awareness of or access to resources available to their child and family specific to NDD-MC), quality of life, support in care planning and management (and resulting social and financial and mental health impacts), and care integration experiences.

#### Case-study

Case studies are ideal to examine the impact of environmental factors on project and policy outcomes (38). A critical component of case study research is defining the case (21). In this paper, cases refer to families with children with NDD-MC enrolled in the NDD-CC project who consented to participate in this research. This study used an embedded case study design given that we had identified *a priori* three distinct subunits of analysis: resource use, quality of life, and care integration. The identification of these subunits was based on the NDD-CC project’s protocol for families, and they are aligned with the selection of the quantitative measures. Bergman (39) suggests that quantitative and qualitative strands should focus on similar thematic areas to avoid data integration challenges (39). Multiple cases were selected to reflect the highly individualized needs of children with NDD-MC and the variability of results observed in the quantitative strand. A single-case study design is insufficient to capture the complexities of this cohort.

### Case selection

**Figure 4:**
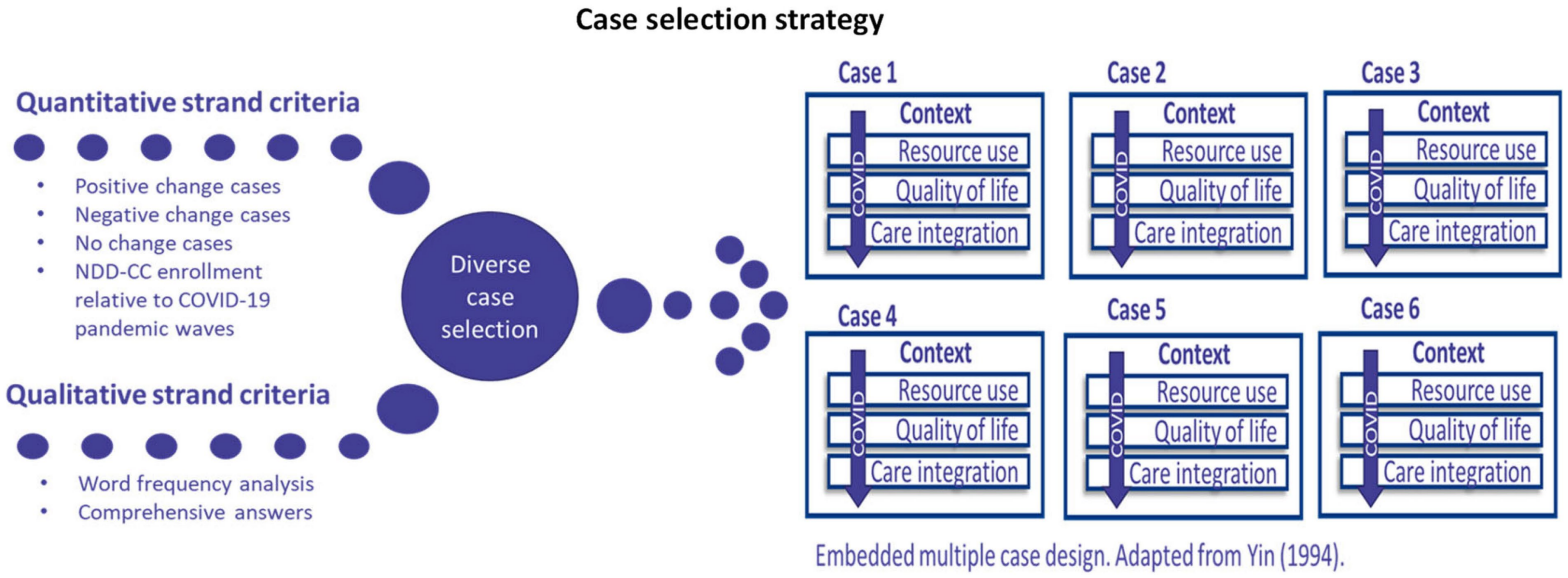
A description of the case selection strategy. Embedded multiple case design adapted from Yin (21).

Cases were selected using the diverse case selection strategy (Figure 4) drawing from the broader qualitative cohort. Diverse case selection refers to integrating cases, which are representative of the range of results observed within a given sample (40). The diverse case method captured the variability of results of the NDD-CC project on our study cohort (40). Representing the full range of results is of particular importance in this study given our cohort’s diverse demographic characteristics (varying levels of medical complexity, variability in NDD diagnosis, age range, income level, etc.). A four-phase approach was undertaken to identify case studies.

First, caregivers must have completed the pre- and post- quantitative surveys and the qualitative semi-structured interviews to be considered in the case study component. Initially, 18 participants fit these criteria.

**Figure 5:**
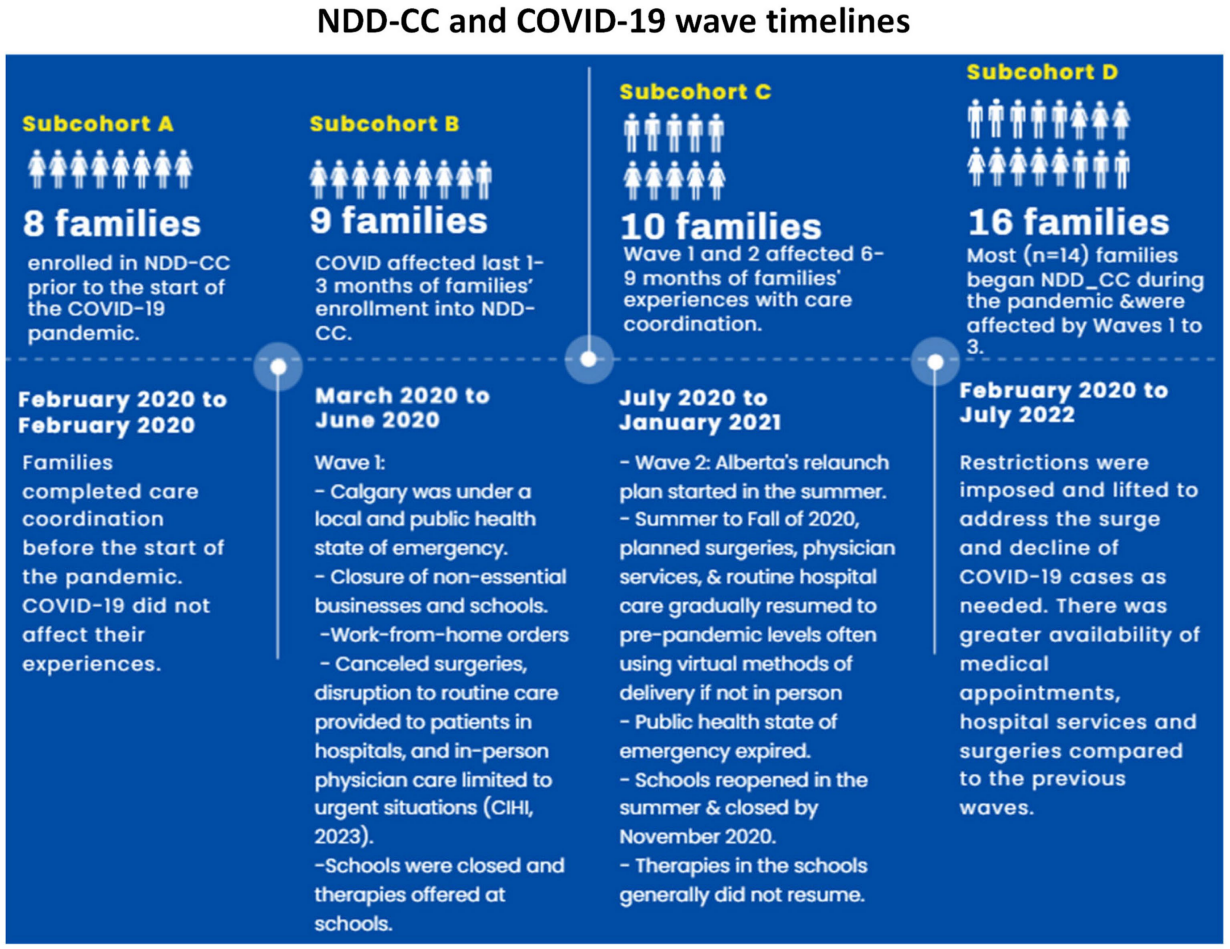
A timeline on the NDD-CC recruitment relative to the different COVID-19 waves.

Second, the research team analyzed the results from the quantitative strand to inform case selection. To represent the variance of the impact of the NDD-CC project on families, researchers grouped participants in the categories described below:

- Positive change case(s): this refers to participants who reported improvements in quantitative outcome measures from baseline to 12-months.
- Negative change case(s): these cases are composed of participants whose 12-months survey results are lower in relation to their baseline survey results.
- No change case(s): this captured participants with similar baseline and 12-month survey results.
- COVID-19 (Figure 5): to ensure that the influences of the COVID-19 pandemic on NDD-CC project were captured, case selection included participants recruited during the different waves of the pandemic.

Next, researchers reviewed the interview transcripts from participants in each of these groupings to identify cases to enhance our understanding of the impact of the NDD-CC project. Of the 18 participants who completed pre-post surveys and qualitative interviews, 11 referenced care integration, resource use, and quality of life domains in their interviews. The 11 transcripts were analyzed using word frequency analysis (references to key concepts of care integration, quality of life, and resource use) (Table 3) and comprehensive answers.

**Table 3:** Qualitative interviews and frequency of parent codes.

**ID**	**Quantitative notes**	**Qualitative notes**			
		**Care integration**	**Quality of life**	**Resource use**	**Total**
P01	Complexity 2, sub B, no change in CarerQoL, decrease in PSI and service usage.	1	0	0	1
P02	Complexity 2, sub D, high baseline CarerQoL, no 12 m PSI, increased services accessed.	0	0	0	0
P03	Complexity 2, sub A, decrease in CarerQoL, increase in services accessed.	0	0	0	0
P04	Complexity 3, sub D, slight decrease in CarerQoL and PSI, no reported change in services accessed.	0	2	0	2
P05	Complexity 2, sub A, minimal change in CarerQoL/PSI/services accessed.	0	1	0	1
P06	Did not complete 12-month RUQ	1	1	1	3
P07	Complexity 2, sub C, increase in CarerQoL and PSI, decrease in services accessed.	0	0	0	0
P08	Complexity 1, sub B, decrease in CarerQoL, increase in PSI, increase in services accessed.	0	1	0	1
P09	Complexity 3, sub D, large decrease in CarerQoL and increase in PSI, decrease in services accessed.	1	0	1	2
P10	Complexity 3, sub D, decrease in CarerQoL and increase in PSI, decrease in services accessed.	2	1	1	4
P11	Complexity 3, sub D, increase in CarerQoL and PSI, minimal change in services accessed.	4	0	0	4
P12	Complexity 2, sub A, no change in CarerQoL, decrease in PSI, increase in services accessed.	3	1	1	5
P13	Complexity 2, sub C, large increase in CarerQoL (42–71), decrease in PSI and services accessed.	0	2	1	3
P14	Complexity 2, sub B, increase in CarerQoL and PSI, large increase in services accessed.	0	1	1	2
P15	Complexity 3, sub A, large increase in CarerQoL (17–69) and services accessed.	0	0	1	1
P16	Complexity 3, sub D, high CarerQoL score, increase in PSI, decrease in services accessed.	0	1	0	1
P17	Complexity 3, sub D, large increase in CarerQoL (45–81), decrease in services accessed.	0	3	2	5
P18	Complexity 3, sub C, large increase in CarerQoL (36–76), decrease in PSI, no change in services accessed.	0	1	0	1
P19	Complexity 2, sub C, increase in CarerQoL, decrease in PSI, increase in services accessed.	2	1	0	3

Finally, four participants from each of the groupings in the quantitative strand who provided comprehensive answers in the qualitative semi-structured interviews were selected for the case study component. Case studies are summarized in the Supplementary Appendix.

The evaluation frameworks and theoretical propositions (22) were the basis of the overall analytical strategy. We used a multilevel model variant of the triangulation mixed methods design. The quantitative and qualitative findings were analyzed (described below) and then merged at the interpretation and analysis stages based on the evaluation framework and theoretical propositions (18).

### Quantitative analysis

#### Administrative data analysis

Statistical analyses were conducted with STATA (version 17.0). Descriptive statistics were conducted for the sample. T-tests and Wilcoxon signed-rank test were performed for sample means and medians of baseline and 12-month data.

#### Survey data analysis

The research team conducted descriptive and summary statistics with Microsoft Excel and STATA.

### Qualitative analysis

#### Semi-structured interview analysis

Research interviews with participants were transcribed verbatim. Findings were analyzed into thematic structures and codes and developed inductively (41). Data was organized and stored on NVivo12 software. A codebook was created and clarified by the research and clinical team weekly. To ensure rigor throughout the research process, researchers practiced reflexivity, sought diversity in perspectives and experience, tracked decisions, and sought input from the research and clinical teams.

#### Case study analysis

**Figure 6:**
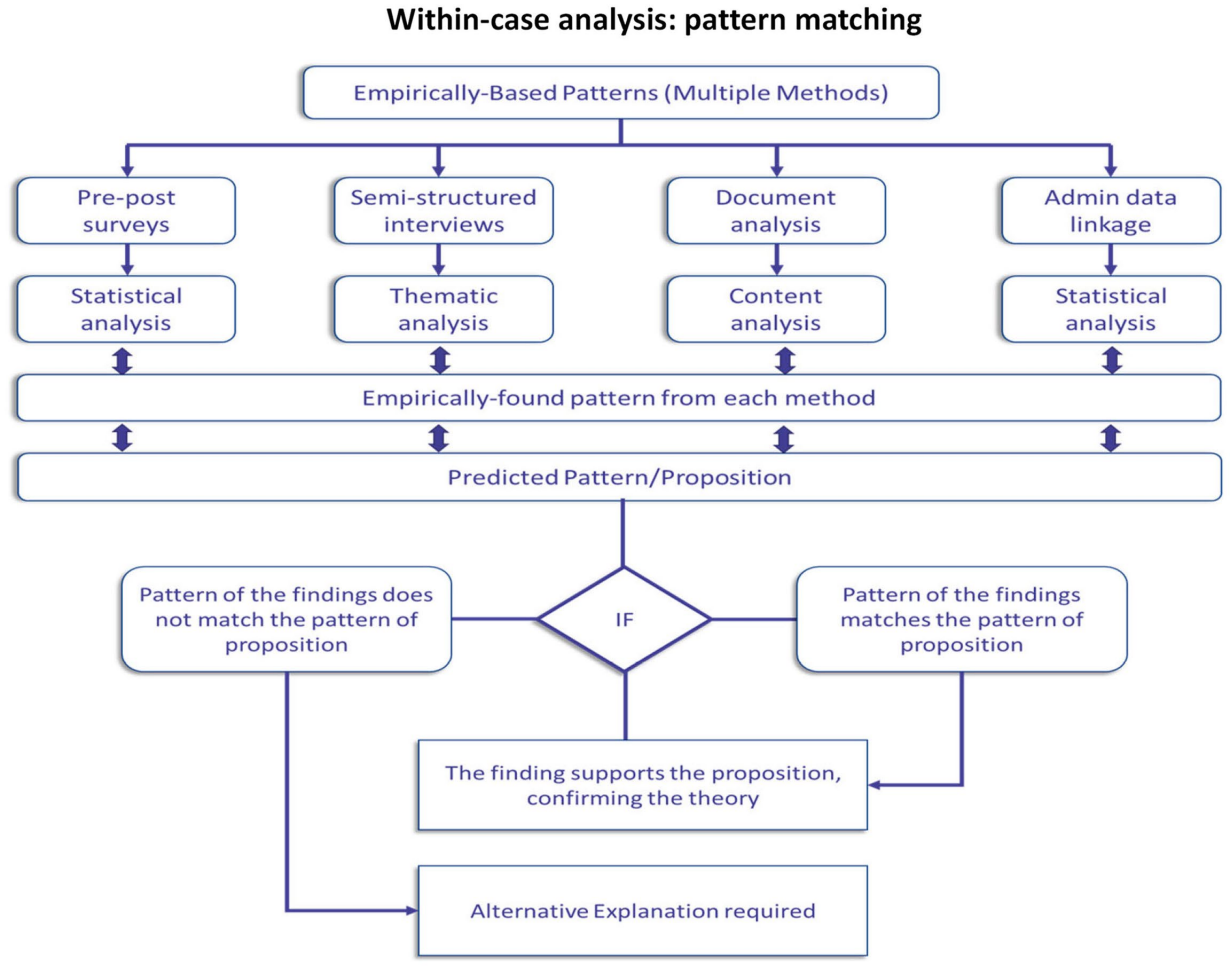
Within-case analysis: pattern-matching methodology. Adapted from (Almutairi et al. (42).

**Figure 7:**
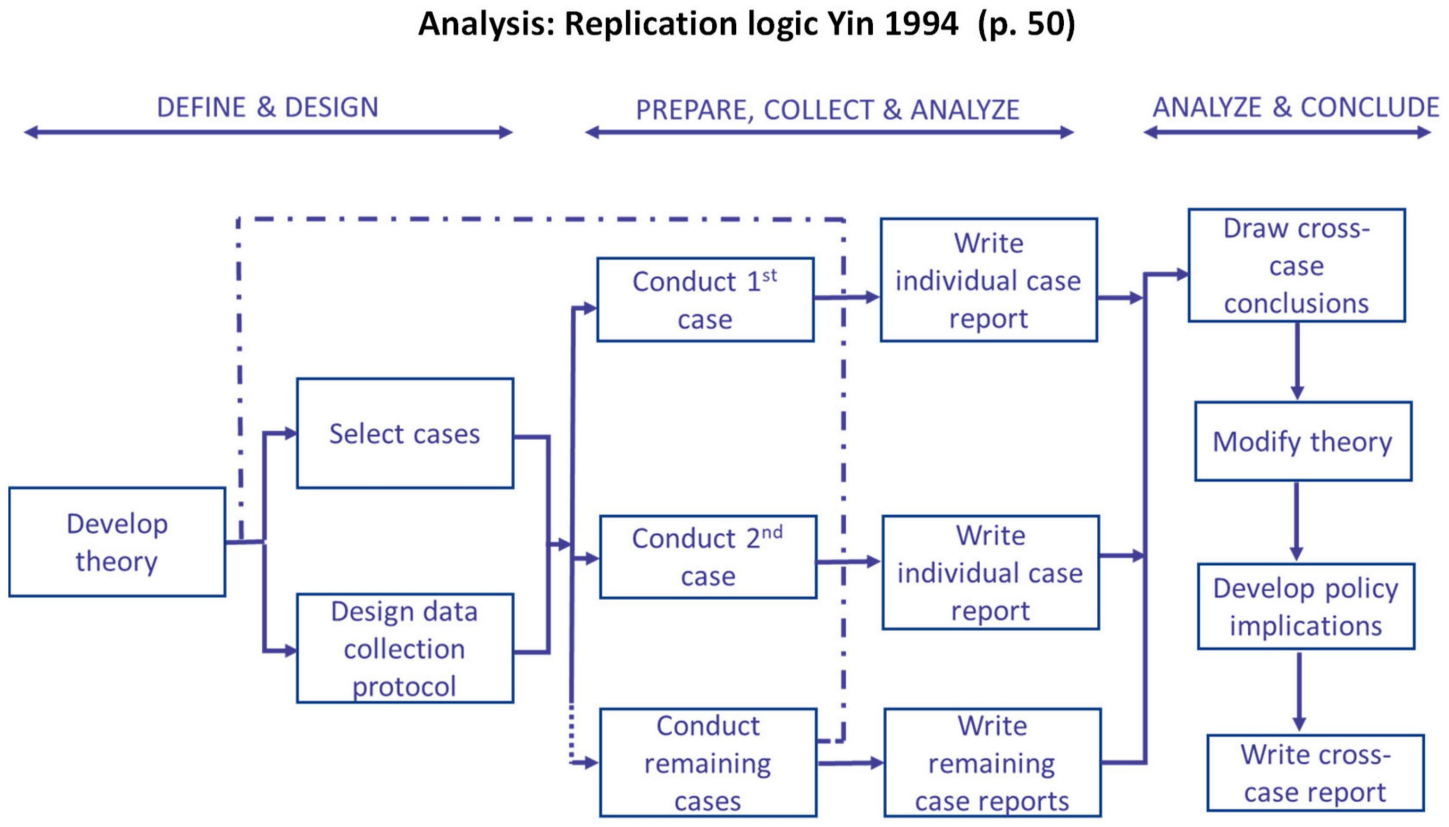
An overview of the replication logic applied to this study adapted from Yin (21).

Using a pattern-matching methodology (Figure 6), we analyzed the case study evidence for each of the three domains. We began our analysis within-case; at this level, we evaluated the findings to test the applicability of theoretical propositions in explaining the changes families observed after participating in the NDD-CC project. The pattern-matching methodology allowed us to establish trustworthiness of findings. Using a replication logic (Figure 7) once all cases were analyzed individually, researchers conducted a cross-case analysis. By applying the replication logic, using the same criteria and procedures to prepare, collect, and analyze within-case data we were able to establish external validity (22).

## Results

### Demographics

#### Quantitative cohort

**Figure 8:**
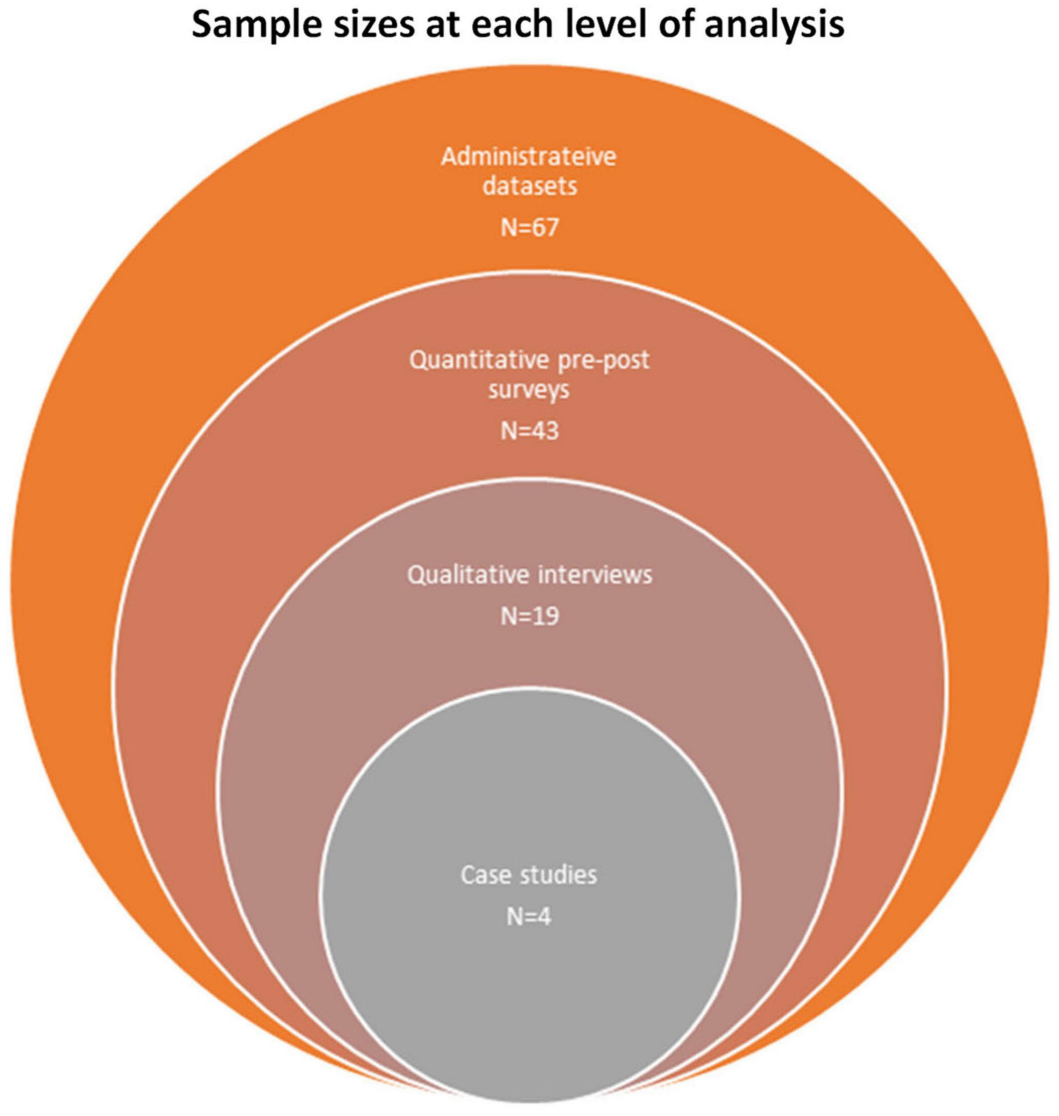
An overview of the sample size for each data source.

The study cohort consisted of 67 families in CC who completed baseline interviews between December 2018 and February 2021. Figure 8 describes the sample for each data source. The linked data covered the period of December 2017 to February 2022, spanning 1 year before and after the first and last baseline interview, respectively. It is important to note that due to the rolling recruitment strategy, families completed pre-post surveys during the different waves of the pandemic (Figure 5). Of the 67 caregivers who met the eligibility criteria, 62 provided baseline demographic information on their children with NDDs and household (Table 4). We obtained data on the ages, level of complexity, and primary NDD diagnosis from the care coordinators on the five families who did not complete the demographic survey. Of this sample of 67, 43 families completed the RUQ and the CarerQoL-7D questionnaires, 34 completed the PSI-4-SF, and 25 completed PICS.

**Table 4:** The demographic profile of children with NDDs, their caregivers, and household.

	**Quantitative sample (*N* = 67)**		**Qualitative sample (*N* = 19)**	
**Variable**	**Frequency (*N*)**	**Percentage (%)**	**Frequency (*N*)**	**Percentage (%)**
*Child characteristics*				
Medical complexity				
1 - Biomedical/systemic complexities	5	7%	1	5%
2 - Biomedical/systemic/moderate psychosocial complexities	41	61%	9	47%
3 - Biomedical/systemic/significant psychosocial complexities	16	24%	9	47%
Not yet determined	5	7%	0	0%
*Age group*			6 to 19 years (x̄=14 years)	
0–5 years	8	12%	1	5%
6–14 years	48	72%	16	84.%
15–17 years	9	13%	1	5%
18 and over	2	3%	1	5.%
*Intake diagnosis*				
ADHD only	30	45%	5	26%
ASD only	18	27%	3	16%
ADHD and ASD	19	28%	10	47%
*Co-occurring disabilities in addition to ADHD only, ASD only, or ASD and ADHD diagnosis*				
Yes	43	64.18%	12	63%
No	19	28.36%	7	37%
Missing data	5	7.46%	N/A	N/A
*Child’s gender*				
Male	38	57%	14	74%
Female	22	33%	5	26%
Non-binary	1	1%	0	0
Transgender male	1	1%	0	0
Missing data	5	7%	0	0
*Caregiver characteristics*				
*Caregiver gender*				
Male	10	15%	5	26%
Female	52	78%	14	74%
Missing data	5	7%	0	0
*Marital status*				
Single (never married)	13	19%	3	16%
Married	42	63%	13	68%
Common law	2	3%	1	5%
Separated	1	1%	0	0%
Divorced	2	3%	1	5%
Widowed	2	3%	1	5%
Missing data	5	7%	0	0
*Relationship to child*				
Mother	53	79%	13	68%
Father	9	13%	4	21%
Foster mom	1	1%	0	0
Grandmother	4	6%	2	11%
*Household characteristics*				
*Number of caregivers in the household over age 18 years*				
1	14	21%	4	21%
2	40	60%	13	68%
3	5	7%	2	11%
4	3	4%	0	0
Missing data	5	7%	0	0
*Important life events and changes in the past 12 months*				
Yes	27	40.3%	9	47%
No	35	52.2%	10	53%
Missing data	5	7.5%	9	47%

##### Children

There were more male (57%) than female (33%) children enrolled in the NDD-CC project; 1% were transgender, and 1% were non-binary. Most children (45%) only had ADHD, followed by 27% who only had ASD, and 28% who had both ADHD and ASD. In addition to the ADHD only, ASD only or ADHD and ASD diagnosis, over 60% of children had multiple co-occurring chronic health conditions. Over 70% of our sample lived in a household with two or more caregivers.

##### Caregivers

The quantitative pre-post surveys were mostly completed by female caregivers (78%). Over 80% of our respondents were parents, 6% were grandmothers, and 1% were foster parents. Forty percent of families surveyed were affected by significant life changes in the 12-months prior to enrolling in NDD-CC, including separation, custody changes, job loss, change of residence, and changes in children’s NDD diagnosis.

#### Qualitative sub-cohort

Maximum variation sampling was used to acquire diversity in the sample; 19 caregivers were selected and interviewed drawing from the larger quantitative sample. The majority of caregivers were mothers (68%), 21% fathers, and 11% grandparents or guardians. Most families had one child enrolled in the NDD-CC project and 16% had two children enrolled. All caregivers identified their children as having medical complexity; 47% of the children had both ADHD and ASD. The average age of children was 14 years with a range of 6–18 years. Most children were male with parents identifying their children as either male or female. Table 4 provides the demographics of this population from the larger sample of caregivers. Similarly, to the quantitative surveys, caregivers completed the qualitative interviews at different stages of the pandemic (Figure 5).

### Analysis

In this section, we describe findings from the analysis of satisfaction, function, clinical, and costs of care domains to assess the impact of the NDD-CC project (Table 5).

**Table 5:** Adapted outcomes and needed measures framework.

Dimension of value	Source	Outcome	
		**Baseline**	**12 Months**
Satisfaction			
Reduce unmet needs	PICS: In the past 12 months, has your child had emotional, developmental, or behavioral problems for which he/she received treatment or therapy?	Yes: 53%	Yes: 56%
		No: 47%	No: 44%
Function			
Ease of access to resource information	PICS: How often did you have difficulties or delays getting medical services for your child because you had trouble getting the information you needed?	Little/no difficulties: 57%	Little/no difficulties: 56%
		Moderate difficulties: 28%	Moderate difficulties: 28%
		High difficulties: 18%	High difficulties: 16%
Achieve self-management skills	PICS: How often has someone on your child’s care team given you resources you needed so that your family could be more independent in caring for your child?	Little/no resources: 38%	Little/no resource: 8%
		Moderate resources: 36%	Moderate resources: 24%
		A lot of resources: 26%	A lot of resources: 64%
Increase functional abilities	EQ-5D-Y: No/some/a lot of problems doing usual activities	No: 16.28% Some: 53.49% A lot: 30.23%	No: 32.56% Some: 39.53% A lot: 27.91%
	RUQ: In the last 12 months did your child attend school including homeschool?	Average VAS: 61	Average VAS: 65
Clinical			
Increase measures of health	EQ-5D-Y: We would like to know how good or bad you think the child’s health is TODAY.	Average VAS: 61	Average VAS: 65
Costs of care			
Reduce emergency department visits	Admin data on ED visits	91 ED visits	62 ED visits
Reduce hospitalizations/hospital days	Admin data on hospitalizations	Total inpatient length of stay: 390 days	Total inpatient length of stay: 185 days
Reduce repeat data gathering by providers	PICS: How often have you had to repeat information about important events in your child’s life or important details about your child’s health that you thought care team members should have known?	Little/no repetition: 40%	Little/no repetition: 20%
		Moderate repetition: 24%	Moderate repetition: 40%
		A lot of repetition: 36%	A lot of repetition: 40%

### Broader economic, policy, social, and environmental influences

Theoretical proposition: Equipping caregivers with resource information specific to their children’s NDDs enables families to access appropriate resources and improves management of chronic health condition (12). In assessing this proposition, we describe impacts of NDD-CC on health service utilization, NDD service utilization, and out of pocket costs by integrating data on ED visits and hospitalizations.

### Quantitative findings: broader economic, policy, social, and environmental influences

#### Emergency department visits, inpatient stays, and physician claims

Among those who had Emergency Dept. (ED) visits, there was a reduction in ED visits after 1 year in NDD-CC, on average. Twenty-seven children in the cohort had ED visits; these totaled 91 and 62 visits at baseline and 12-month, respectively, a 31.9% reduction. The sample mean was 1.4 ED visits (SD 2.3) at baseline and decreased to 0.9 visits (SD 1.6) at 12-month follow-up. Nineteen and 14 children had two or more ED visits at baseline and 12-month, respectively.

Reduced ED visits likely translated to reduced acute care costs. The total sample ED physician claims costs were estimated to be $27,435.93 at baseline, decreasing to $16,422.56 at 12-month, a 40.1% reduction. The maximum estimated physician ED claims’ cost per child were $3679.74 and $2025.51 at baseline and 12-month, respectively.

For those who had hospital stays, the total length of stay (LOS) in hospital was reduced. Fifteen children had 33 inpatient stays with a total LOS of 390 days at baseline, which decreased to 10 children and 23 inpatient stays with a total LOS of 185 days at 12-month. The sample mean was 0.5 stays (SD 1.1) and 0.3 stays (SD 0.9) at baseline and 12-month, respectively. The maximum LOS per child was 97 and 42 days at baseline and 12-month, respectively. The observed sample reductions for the number of inpatient stays and LOS were 33.3% and 52.6%, respectively. The Wilcoxon signed-rank test indicated a statistically significant difference in the median LOS of the sample cohort (value of *p* = 0.002).

**Figure 9:**
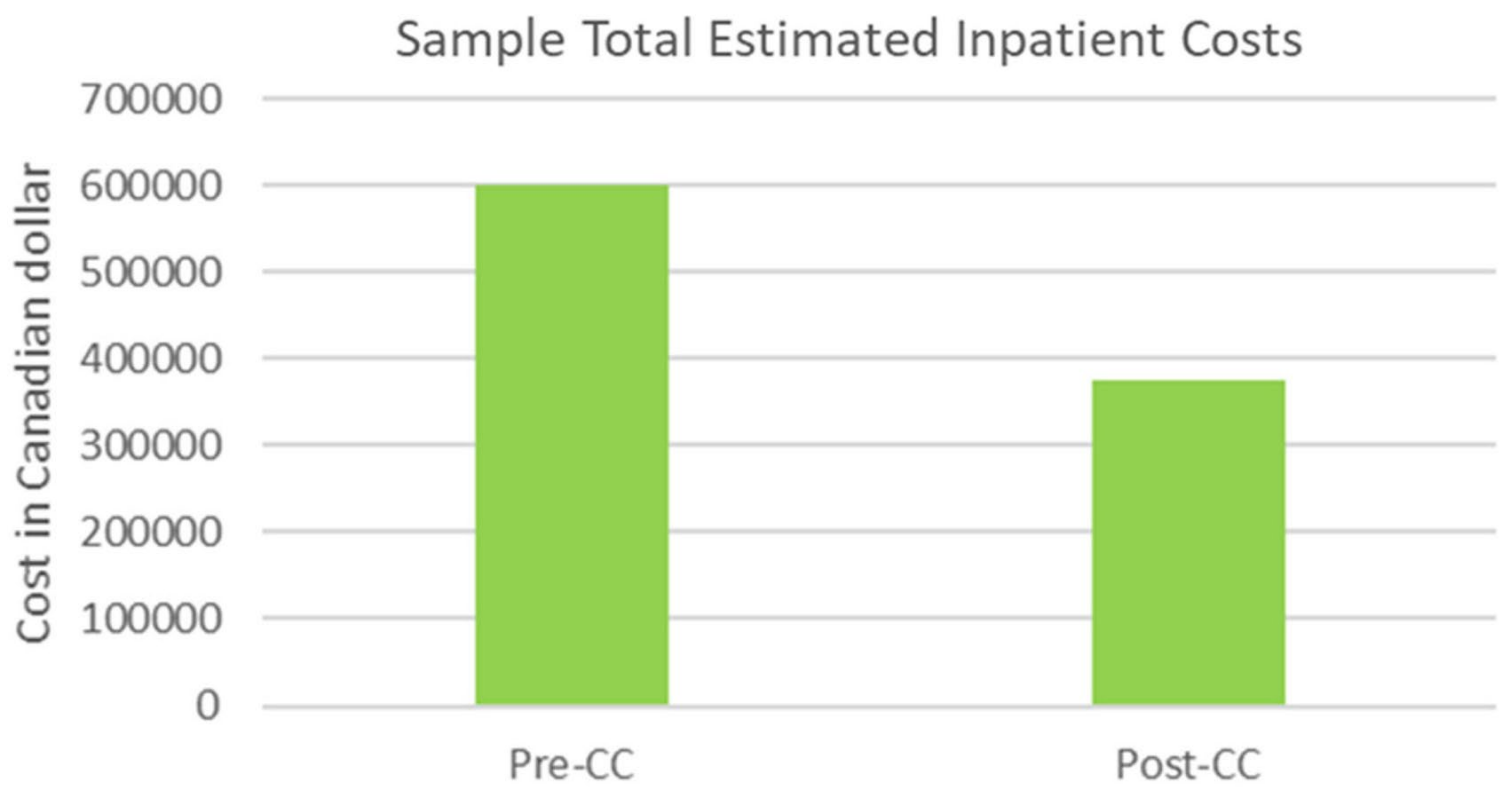
Sample estimated inpatient costs.

This reduction in LOS also resulted in cost savings. At baseline, the total sample inpatient costs were estimated to be $601,221.6 (Figure 9). The average and median RIW were 2.04 and 2.31, respectively, with the range between 0.36 to 12.49. Twenty-two (66.7%) out of 33 inpatient cases had RIW values greater than 1. Sixteen (48.5%) cases were coded 709 for childhood/adolescent development disorder. At 12-month post-CC, the inpatient costs were estimated to be $375,469.20. The average and median RIW were 1.65 and 1.43, respectively, with the range between 0.31 to 3.68. Nine (39.1%) out of 23 cases were coded 709 and 14 (60.9%) had RIW values greater than one.

An estimated reduction of $225,752.41 (37.5%) was observed in inpatient costs between one year pre- and post-CC. All inpatient cases coded 709 had RIW values greater than two, illustrating the relative higher resource utilization of this clinical population.

A reduction in physician claims costs was also observed, however there was a great deal of variability in the population. At baseline, physician claims costs per child were estimated to range from $151.59 to $31,192.04, with an average and median of $4039.87 (SD 6133.90) and $2107.83, respectively. The number of days children received health care services ranged from 1 to 105 days per child, with an average and median of 20.6 days (SD 19.7) and 14 days, respectively. The number of different health care provider settings each child visited ranged from one to ten, with an average and median of 4.3 (SD 2.1) and 4, respectively.

At 12-month, physician claims costs per child were estimated to range from $155.70 to $19,428.50, with an average and median of $2916.41 (SD 3687.47) and $1684.65, respectively. The number of days children received health care services ranged from 2 to 94 days per child, with an average and median of 18.6 days (SD 18.6) and 13 days, respectively. The number of different health care provider settings each child visited ranged from one to twelve, with an average and median of 3.9 (SD 2.2) and 3, respectively.

A reduction of 27.8 and 20.1% in average and median physician claims cost per child was observed, respectively; *p*-values for *t*-test and Wilcoxon signed-rank test of means and medians were 0.06 and 0.02, respectively (null hypothesis: no difference in mean/median physician claims per child at baseline and 12-month). A two-day or 10.8% reduction in health care claims days was also observed, which may suggest a reduction in requirements for time off for caregiving needs.

#### NDD services – resource use questionnaire

Access to NDD services did not change substantially following NDD-CC. Reported NDD service utilization ranged from 0 to 4 services per family and 0 to 5 services per family at baseline and 12-month, respectively. At baseline and 12-month, 21 and 19 families, respectively, reported no service utilization. Fourteen families cited an increase in service use, 11 reported a decrease, and 18 reported no change. The sample change in service utilization was +0.14 (SD 0.14, *p* > 0.05).

#### Therapy, educational supports, out of pocket costs

Average out of pocket (OOP) expenses per family were $2732.83 (SD $3916.39) and $1894.70 (SD 3024.17) at baseline and 12-month, respectively. Reductions in OOP expenses were observed in 25 families, while 18 families reported increases or no change. COVID-19 may have contributed to lower OOP with reductions in available services during that time period (Figure 5).

#### Reduce repeat data gathering by providers

The percentage of participants who reported repeating information about their child with care team members increased from baseline to 12-months. At baseline, almost half (40%) of caregivers reported little/no circumstances in which they were required to repeat information to care teams. By 12-months, only 20% of caregivers were in this category, with most (80%) reporting moderate or a lot of repeated information sharing.

### Qualitative findings: broader economic, policy, social, and environmental influences

Care coordinators are referred to as CC1 or CC2 in the findings. Parents are referred to as P with an identifier number.

Prior to involvement with NDD-CC, many parents said they did not know what resources were available to them in the community to manage medical, behavioral, and educational needs for their children. One parent noted,

…you are also lost. You have no idea what you should ask or what you should be concerned about. You do not know what to ask for, if you have no idea what’s available or if you know what’s available, you do not even know what you are entitled to (P17).

Even though parents were working with programs such as provincial government disability support such as Family Supports for Children with Disabilities (FSCD) they did not know what resources they could access for their children. The same parent added more,

So when you get signed up with FSCD….we were never told about any of this stuff….you have to try and figure it out on your own…There was never, um, a cut and dry thing where there was, here are the services that are available, um, if your child qualifies, there’s just nothing. It’s just kinda like, here’s the contract and then they basically hope that they will not hear from you again, right? Well with CC1, she said, do you have this? Do you have this? Do you have this? (P17).

Parents indicated that care coordinators navigated systems and sectors and found the resources that matched the issues the family was experiencing. One parent said, “she (CC1) can kinda put you in touch with the right people and, and actually knows what is out there.” (P14) and another parent said, “it’s just nice to have a person like that that can really get a full picture of your family and, and recommend things and, and then help you get there.” (P13). Care coordinators assisted families to get a variety of resources from in home support to food support. “they talk about, for example, some ideas to improve between, you know, in-home support and, eh, behavioral therapy.” (P10). And another parent shared, “She, um, was good at getting us, uh, food hampers, Christmas hampers, and getting them on the Christmas list for Santa Claus. She did all of that with us.” (P12).

Once parents knew what resources were available to them, they felt empowered to advocate for what they needed. One parent said,

I did not even know a lot of the services existed prior to her. And, now that I have that better understanding I’m able like I always advocated prior but with knowing a lot more of what were entitled to with the help CC1 pushed that even more (P06).

### Case study: broader economic, policy, social, and environmental influences

Most of the propositions were met with the introduction of CC as illustrated in the case studies. Families had access to resources specific to their child’s needs. They also received interventions, which were adapted to meet the child’s medical, social, and educational needs. The case study evidence demonstrated how the NDD-CC tailored its support to address individualized needs:

- P12 received support in accessing after-school programs, bus tickets, food hampers, Christmas hampers, and assistance in completing income tax forms.
- P10: Community-based, in-home, behavioral supports, and out-of-home placements for medical and mental health challenges were provided.
- P17 was connected to community-based resources, received support for a new school placement, accessing FSCD, Federal tax benefits, and the child development center.
- We were unable to assess the full extent of applicability of the costs of care proposition on P19 due to the lack of resources available from COVID-19 mitigation policies (Figure 5).

In addition, caregivers discussed the role of accessing NDD-specific resources in the management of children’s NDDs, confirming another component of this study proposition. The P12 case study proposed an expansion to our theoretical proposition. This case demonstrated the duality of the benefits of access to appropriate NDD-resources and the improvement in caregiver quality of life and meeting NDD-MC needs. Moreover, in describing their experiences with service navigation, caregivers (P12, P10, and P17) described feeling supported by their care coordinators in navigating the complex network of NDD services.

### Community environments, networks, and formal services

Theoretical proposition: The quality of care integration experienced by families with children with NDD-MC is determined by the degree of family engagement with care teams in care planning for their children with NDD-MC (12).

#### Care integration measures from survey data

**Figure 10:**
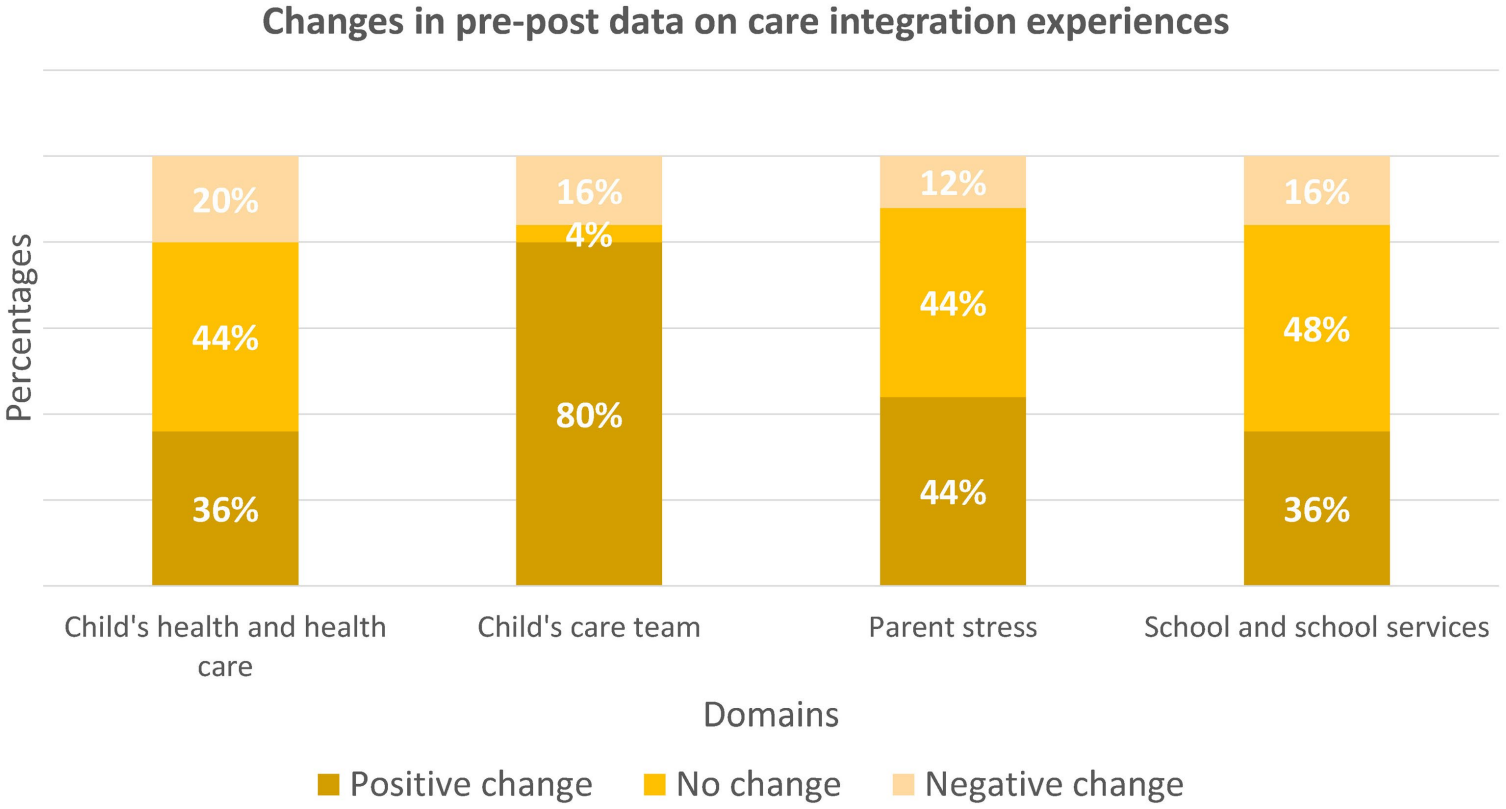
An overview of the changes in pre-post data on care integration experiences.

At baseline and 12-months, 25 caregivers completed PICS. In addition to grouping responses into low performing, medium performing, and high performing based on level of difficulties they reported, we also discussed changes in pre-post results. The most significant improvements were reported in the child’s care team and parental stress constructs (Figure 10).

- Parenting stress. At baseline, 88% of caregivers reported the highest level of stress, by 12-months, only 64% were in this category. Caregivers reported that their care teams more frequently addressed the aspects of their lives which caused them stress and the impact of their children’s health on the family quality of life. Caregivers agreed that integrating family quality of life in addition to addressing children with NDD-MC’s health contributed to lowering parenting stress.
- School and school services. Caregivers continued to grapple with challenges with school-related services. At baseline, none of the families reported high quality of service in the school setting; this trend continued at 12-months. In addition, 48% of families reported no change in the level of difficulty experienced in accessing school support and 16% reported a negative change. Despite this, the number of families reporting the highest level of difficulty reduced from 72% at baseline to 56% at 12-months. Caregivers reported that their children were able to access educational support more easily because of NDD-CC.
- Child’s care team is the domain where caregivers reported the most significant improvements. Eighty percent of caregivers surveyed reported a positive change in the quality of support provided by their care teams. Caregivers reported improved communications with their care teams, greater parental involvement in care planning for their children with NDD-MC, and improved coordination among the different care team members. At baseline, 40% of caregivers reported the highest level of difficulty with their care teams (low-performing category) and this reduced to 12% at 12-months. On the other hand, the proportion of families with high-performing care teams remained unchanged at 60% across the two time-points.
- Child health and healthcare. Most respondents reported no changes (44%) or positive changes (36%) in accessing needed medical services for their children with NDD-MC. The percentage of families with little to no difficulties in accessing medical services remained unchanged: 40% at baseline and 12-months. Conversely, with 44% of families at baseline and 36% of families at 12-months who were in the low-performing category, this was not the case. Caregivers in the low-performing category reported challenges obtaining needed information to access medical services. In addition, these families reported that waiting lists and backlogs caused significant delays in accessing services.

### Qualitative findings: community environments, networks, and formal services

Caregivers shared that care coordinators provided comprehensive care specific to their child and family’s needs and anticipated what could be needed in the future. As one caregiver shared, “We get lost in the details and she [CC1] sees the bigger picture” (P11) and this parent explained further, “[She] looks at the different aspects of our case and tries to figure out where we might need help and-... what, uh, what we actually might need to be doing next.” (P11). There was a sense of knowing what was required to navigate and integrate between and within sectors explained by a caregiver,

….part of that integration was she being able to, you know, connect us, make sure that we are getting the best care. Make sure that there is a follow up, make sure that everything is, you know, working, all the other parts are moving (P01).

Care coordinators had a broad understanding of health and managing care needs and the need for integration of care with schools, health and disability support as important sectors influencing health. One caregiver described the care integration for her child with NDD.

She organized this big meeting with the psychiatrist, and the community pediatrician, and the mental health clinic. This is a connection between families, hospital, you know, um, health centers so... they talk about, for example, some ideas to improve between, you know, in-home support and, eh, behavioral therapy (P10).

Another parent shared how care integration involved multiple health and disability providers to meet family care needs.

She organized a meeting…all the team members were there. So the psychologist. The speech therapist. The occupational therapist. The... I do not remember if the physiotherapist was there in person or not that time. Uh, she might’ve called in. And, uh, the FSCD worker, our support worker looking at the different aspects of our case and trying to figure out where we might need help and-... what, uh, what we actually might need to be doing next.… (P11).

The importance of including the school sector in influencing health and outcomes and therefore working with school providers was a particular nuance of the NDD-CC project. As one parent stated, “she organized, … this, eh, meeting, you know, with the school….people from the school, Children’s Village, you know, I remember eh, [child’s name] teacher and also the principal, the school principal” (P10).

Another parent shared the integration of the child’s diagnosis and behaviors that were considered challenging in the school setting was helpful. “She went to medical appointments with us. She came into the school meetings with us. She told them all about their diagnosis and what the circumstances they are on, and, um, their, their behaviors and everything else.” (P12). When asked what difference did this make the caregiver replied, “everything started going smooth.” (P12).

Integration of care within schools was also mentioned by another caregiver including the need to move schools to meet the needs of her child:

I finally said I had enough. I pulled them both, both my kids out of the school. So, she [CC2] coordinated and organized a huge meeting with the public school board... and it was pretty much CC2, the assistant principal from the old school, and the new principal from the new school that got him into the new program (P19).

In addition, another parent discussed the integration of disability support from CC through the FSCD program.

She was the one, she was there for our meetings with FSCD and everything. She came to a couple doctor’s appointments and really advocated and especially with the FSCD meeting because I did not know what I was talking about. I did not know what we could ask for. And, so, she really, she was that voice that really got us what we needed and in the end our FSCD worker was like, yeah, did not even think of it, like you guys should have this and they got it. So, without her we would never have had all the at-home supports (P06).

Care integration also involved coordinators helping caregivers understand what happened at the multiple care meetings they attended for their child.

She was there and on our behalf if we did not understand something she was there to help us understand it, and as well speak on our behalf to inform the school and to know more about kids and understand them a bit more. She always explained down to my level to help me understand (P12).

Care integration came with challenges in advocating for the needs of the families with systems with few resources. One caregiver discussed how she felt protected with CC.

Well, just before care coordination I was only going in there with these doctors and being told that that’s not possible, or it’s not within funding, or anything else, and then when CC2 got involved she started ripping the layers of the onion apart (P19).

There was also a realization that care integration was a necessary support for families who could not do it alone anymore. Two parents shared their perspectives,

It wasn’t that we necessarily understood more, it’s just that we came to the realization that we just cannot really do it-... alone, you know? Like, as parents, which was a horrible realization to have to come to, but-... it was the case (P09).

And another shared, “we are also too enmeshed in it and we are also burned out. Sometimes we do not- we do not ask the right questions or we are not seeing things as they are happening or it is- it is really helpful to have another person help- help us navigate” (P11).

In addition, caregivers also discussed the impacts of the pandemic-related restrictions on access to services and family quality of life. Caregivers struggled to access needed resources to manage their children’s NDD-MC, stress and burden associated with the lack of support from formal services, increased caregiving responsibilities, and the impact of their children’s inability to socialize with peers. Despite this, caregivers mentioned that NDD-CC played an important role in supporting families during the pandemic. See Currie et al., 2023 for further discussion of these findings.

### Case studies: community environments, networks, and formal services

In the case studies, coordinators assisted with care integration with providers to determine a plan of care for the child and family. The exception being when care coordinators referred families to resources or providers which were no longer available or were postponed because of pandemic restrictions. We noted that the strategies to engage families in care integration varied across the four cases.

- P12: This caregiver noted that she had received judgment-free support and advocacy for her child with NDD-MC from the care coordinator. P10, P17, and P19: illustrated that care coordinators were able to stimulate family engagement in care planning by creating discussion forums with various members of the children’s NDD-MC care teams. Families spoke of the role of care coordinators in managing information to reduce miscommunication and ensure clarity on care planning. In addition, these cases demonstrated the role of integrated discussion forums in streamlining a family engagement approach across the different sectors that care for children with NDD-MC.
- P17 and P19: In these cases, the quality of care experienced by families was also in part determined by the presence of the care coordinators. Given that, caregivers shared that care teams communicated more openly with families in the presence of a care coordinator. The presence of a care coordinator also made caregivers more confident to ask questions. Furthermore, P17 illustrated the role of care coordinators in increasing family health literacy and creating opportunities for caregivers to apply this literacy when advocating for their child’s care needs.
- In addition, P19 described the protective factor of care coordinators. This case portrayed care coordinators representing the needs and perspectives of caregivers. Care coordinators voiced the concerns of caregivers to ensure family engagement in care planning decisions, especially in circumstances where caregivers voices were “ignored,” “dismissed,” or “trampled.” Care coordinators provided follow-up caregiver concerns, which were not addressed by care teams.

### Household: function and satisfaction

Theoretical proposition: To improve family quality of life, CC interventions should be flexible to address the changeability of children with NDD-MC’s medical, educational, and social care needs (12).

To measure changes in the function domain, three dimensions were focused on: ease of access to resource information, achieve self-management skills, and increase functional abilities:

- Ease of access to resource information. The degree of difficulty and delays in accessing resource information between baseline and 12-months was almost identical. Despite this, our pre-post findings indicated a slight reduction in the barriers caregivers encountered to access NDD-specific resource information.
- Achieve self-management skills. We noted the most significant improvements in this dimension of value. At baseline, 38% of caregivers reported that their care teams provided them with little to no resources to enable them to care independently for their children with NDD-MC. By 12-months, only 8% of caregivers were in this category, with most (64%) reporting frequent access to resources to independently care for their children with NDD-MC.
- Increase functional abilities. To assess changes to functional abilities we assessed two components. First, caregivers’ perception of the challenges their children encountered in performing daily activities. Findings from the EQ-5D-Y indicated improvements in children’s functional abilities. The number of caregivers who reported that their child was able to perform usual activities (such as playing, going to school, playing sports, etc.) without any problems rose by approximately 16%. Similarly, there was a slight decrease (≈2%) in the number of children who experienced a lot of problems in performing usual activities. Second, we analyzed data on school attendance. At baseline, all children were attending school, by the 12-month mark, 2 children (≈5%) were not attending school, this occurred after the start of the COVID-19 pandemic (Figure 5).

The satisfaction domain of this framework evaluated unmet needs:

- Reduced unmet needs. We noted a slight increase in the percentage of caregivers who reported that their children with NDD-MC were able to access needed services to address emotional, developmental, or behavioral problems. The number of families with children with NDD-MC with access to needed services to manage their children’s needs rose by 3% from baseline to 12-months.

The clinical domain assessed changes to children’s health:

- Increased measures of health. Findings from the EQ-VAS, a measure of caregivers’ perception of their children’s health at the time of survey completion, indicated a slight improvement in children’s health. The EQ-VAS score rose from 61 to 65.

An increase in measures of health is integrated into this framework to assess improvements in the clinical domain.

### Increase measures of health included quality of life measures

#### Quantitative findings: quality of life measures

##### CarerQoL-7D

Forty-three families completed the baseline and 12-month CarerQoL-7D questionnaires. Scores ranged from 17 to 96 and 17.8 to 100 at baseline and 12-month, respectively. At 12-month, one family reported a score of 100. Score changes between baseline and 12-months varied from −36 to +52.3. Twenty-five, 16, and 2 families reported positive change, negative change, and no change, respectively. The mean change in the sample was +6.4 (SD 19.8, *p* < 0.05).

##### PSI-4-SF

Forty-one and 34 families completed the baseline and 12-month PSI-4-SF, respectively; 32 families completed both the baseline and 12-month PSI-4-SF. At baseline, Total Stress (TS) scores ranged from 67 to 149 and 69 to 165 at 12-month. Changes in scores ranged from −42 to +49. Seventeen families reported positive changes (reduction in TS score), and 15 families reported negative changes. The mean change in TS score in the 32 families was −0.28 (SD 19.39, *p* > 0.05).

#### Qualitative findings: household: function and satisfaction

Caregivers spoke of the relentless care needs of children with NDD-MC, with behavioral issues and the impact on everyday life with not being able to anticipate the next crisis. As one parent shared, “it seems like, like I would work for a bit and then a crisis would happen. I know a lot of, um, medical parents have, um, crisis after crisis after crisis and it never stops.” (P17). She elaborated further, “you do not even know what to prepare for-... because you do not know what the next thing is gonna be.” (P17).

There was a sense that NDD-CC was about preparing parents for these crises and what could happen next, even with families experiencing many barriers. A parent shared she felt better prepared for the unexpected challenges that came with having a child with NDD and MC. “I do not know what’s gonna happen, but something is gonna happen. So, so, that kind of a coordination program helped me to, to, to be prepared for something. To be prepared for the sudden changes.” (P08). Care coordinators tried to work with the challenges and get the support that was required.

With a counselor or a social worker, we can vent and they can say, oh, I’m so sorry, but cannot do anything. When I, I spent an hour with CC1 and then she’s like, “okay, I’m gonna do this, this and this.” It’s actual action (P04).

And another parent said, “It was, um, a lot less stressful. I mean, cuz she was up on everything. She was, “Okay. [name of parent], we get an appointment here. [name], we got an appointment here. [name] we gotta do this. [name], we are gotta do that.” (P12).

Parents shared they had difficulties advocating and coordinating for their children alone before NDD-CC and this contributed to feelings of helplessness.

When going to see the professionals, the specialists, um, you already feel very small. You have to fight for your child no matter where you go. I mean, sometimes you get a really good doctor and they help you out and they listen and all those things, but most of the time you do not.... and, um, in these situations, like I felt like I was getting trampled by neurology initially (P17).

There was the sense of feeling supported through the care coordinator’s physical and emotional presence. This was shared by several parents. “And I talked to her [CC1] beforehand about what my concerns were, and it was almost like you have someone on your team.” (P17). And another shared,

I do not think it was anything that she did. I... she just was kind of like my shield, I guess you could say… She was pretty much my shield. Like, when they would ask for meeting I’m like, “Okay, yeah. Um, let me get in touch with CC2 (P19).

Other parents concurred, “just having her there as my support woman. Someone on my side, hey.” (P14) and “someone that, um, is not judging you about things that you need help with, right?” (P17). This support decreased feelings of stress and isolation.

I feel like I have an advocate, which decreases my stress levels. I mean, I’m still stressed, but it decreases my stress levels … I think the care coordination program has impacted the quality of life, because... it gives me an advocate that I did not have before (P04).

And another parent also discussed she felt less isolated,

You feel supported. Um, you feel you have somebody to help you, you are not alone trying to navigate, uh, how frustrating the system is, and, and actually yeah, to have somebody there for you (P05).

Parents spoke of the longer term outcomes of being involved in NDD-CC.

So, she taught me to like stand up for myself and stand up for that and go like, “Okay, I need a break, like it’s okay.” … And so like CC1 gave me that voice to really just be like, “You know what, no. We need this, I need this” (P06).

Other parents spoke of the impact of managing their child’s care needs with the loss of NDD-CC when their time in the program was finished.

How I’m not losing it, I do not know. You’re doing good, you know but like I told her (CC1), I’m so tired of everybody saying, “You’re doing a great job, you are just fantastic, yeah and see you in 3 months…. To have that resource, um, because when she was gone I had nobody (P16).

And another parent discussed the ongoing need for NDD-CC, “you know, there needs to be a support that needs to be ongoing.” (P18).

#### Case studies: household: function and satisfaction

Some families continued to experience high stress levels and poorer quality of life with lack of support in managing changing care needs influenced by the pandemic. The case study findings confirmed the function and satisfaction theoretical proposition. Caregivers described ways in which CC improved their quality of life. In addition, a small number of cases described other factors beyond NDD-CC, which affected their quality of life.

- P10, P12, and P19: These families described how supports provided by the CC specific for their children with NDD-MC, reduced day-to-day stress and consequently improved their quality of life. Conversely, for P17 the inability to access NDD services because of COVID-19 restrictions did not enable this family to receive support in caring for their child with NDD-MC and limited the impact of the NDD-CC project. These limitations caused stress for this family.
- P10, P17, and P19 directly spoke of the consistent support provided by care coordinators in addressing children’s with NDD-MC changing needs. In addition, P19 described the positive impact her family experienced from her coordinator communicating with care teams and creating transition plans to cater to her child’s needs.
- P10 presented the unique challenges of immigrant families in accessing NDD-specific resources. This case presented macro level limitations of immigration and health policies to facilitate access to NDD supports for newcomers in Canada.
- Although P12 did not directly address the changeability of children with NDD-MC needs, this case described the centrality of clear communications in reducing stress and improving quality of life. This case confirmed the function and satisfaction propositions and showed that families see care coordinators as an extension of their own family unit (Tables 1, 2).

## Discussion

Here, we discuss the contributions of our study to the literature, practice, and policy of measuring and implementing CC interventions for families of children with NDD-MC and implications for future research. The following are *key considerations in assessing CC interventions for children with NDD-MC.* By integrating the Center for Community Child Health’s (Platforms) Ecological model and Antonelli et al.’s Outcomes and Needed Measures framework, this study adopted a multidisciplinary, multilevel approach to assess the impact of the NDD-CC intervention on children with NDD-MC and their families (Figure 1). Results from the different care integration, resource use, and quality of life measures used indicated high variability of results across domains and households. Progress was not linear for families; improvements in one area/domain did not have a ripple effect to the other domains. Therefore, these findings emphasize the importance of using an ecological model and a multidisciplinary approach to assess the impact of different system level influences on the outcomes achieved by CC interventions. Despite the proliferation of academic studies highlighting the importance of CC and its recognition in clinical practice, measuring its impact remains challenging (43–49). Overall, existing CC literature for the pediatric population has used contrasting outcome measures and tools, evaluated interventions in different settings (primary care, tertiary care, emergency department-based interventions, etc.), and operated under various funding mechanisms, leading to discussion on the influence of these factors in assessing the impact, scalability, and replicability of interventions for similar populations (43–50). CC studies on pediatric populations with MC are scarce (43), and the heterogeneity of this population has led to mixed results in evaluation studies (44, 47), prompting a lack of clarity on how these interventions should be delivered and assessed (44, 45, 49). Our study which contributes to the expansion of CC research for the pediatric population builds on this foundation of previous literature and emphasizes the importance of adopting a measurement framework at the systems and household level. Capturing system level impacts is critical as indicated by the reduced costs associated with acute care because of the CC intervention, however equally important is incorporating a descriptive approach that account for high variability in patient outcomes (51).

### Impacts of the NDD-CC intervention on resource use and family quality of life

#### The role of CC in reducing costs of care

The relationship between the role of access to resources in improving long-term management of NDD-CC and its subsequent effect on reducing costs of care proposition may be ambiguous to the reader. We deem it necessary to clarify this. First, quantitative findings indicated an overall reduction in ED visits and acute care costs; reduction to ED visits is often the benchmark of successful CC interventions. Existing literature shows a positive correlation between reduced ED visits and access to adequate and consistent support from physicians, specialists, and disability support workers for CMC (50). With increased access to resources, long-term care management of CMC is improved, and the reliance on ED visits reduces.

Second, qualitative findings covered two important aspects. Through adequate needs-based matching, NDD-CC promoted optimal use of resources, reducing avoidable ED visits by giving families access to information, resources, timely and consistent navigation support, and facilitating access to disability support workers, primary care physicians, and other specialists. In addition, our findings allude to the continuously high caregiver burden, which NDD-CC was able to address to some extent, leaving families feeling less isolated and alone, and better able to manage the continuous care needs. The profound social and economic costs of care to family/friend caregivers has been recognized by the Federal government through its Employment and Social Development Canada agency descriptions of the various expressions of caregiver burden, its short and long-term impacts (52). A cost–benefit analysis of the labor and leisure time foregone could paint a more concrete picture of these personal costs; however, that is beyond the scope of this paper.

The observed reductions in ED visits, inpatient stays, and health care service days in our study cohort suggest improvements in coordination of care, translating to cost savings for the health care system. Previous studies also found that CC reduced hospitalizations and costs (53–55). The decreased number of different provider settings and physician claims days could suggest that the children are receiving focused health care services rather than being referred to several providers that may not address their specific needs. Due to the absence of a control group, it is not possible to link the above changes solely to the CC intervention.

### NDD-CC intervention is integral to CMC’s access to physicians and other specialists

Hospitals and physicians account for the largest share of total health care spending in Canada, at approximately 24.3 and 13.6%, respectively, in 2022 (56). While CMC account for less than 1% of the child population, they can account for up to one-third of all pediatric health care spending (57–59). Children with medical complexity have intensive hospital service needs (5), which was illustrated in our cohort’s hospitalizations, with over 60% of cases at baseline and 12-month having RIW values >1 and a significant number of cases having LOS exceeding 10 days.

The reduction in acute care and inpatient costs is congruent with existing literature, providing further evidence on the cost-effectiveness of nurse-driven CC interventions when compared to physician-driven CC (50). Consistent with previous studies (50), our findings illustrated a reduction in ED visits following families’ participation in NDD-CC. Additionally, literature suggests that a reduction in acute illness office visits is one of the benefits of CC interventions (50); however, data from our sample shows an increase in the range of physician and specialist visits from 0 to 43 at baseline to 0 to 107 at 12-months. Although, we do not have access to data r documentation for every physician or specialist visit, in the qualitative interviews, caregivers described increased access to medical services as an important benefit of NDD-CC. Therefore, we hypothesize that a significant portion of these visits was linked to increased access to physician/specialists as part of NDD-CC’s strategy toward improved long-term management of child’s NDD-MC rather than an indication of an increase in acute illness office visits.

#### Isolation and support: significant predictors of caregiver quality of life and parental stress

Aggregate findings from our quantitative surveys showed variable impact on families’ health and quality of life. Qualitative findings highlighted barriers and facilitators likely influencing this variability. Consistent with previous literature (61), caregivers mentioned that parental advocacy facilitated access to services, a skill which they were able to develop with support from care coordinators. However, the need for parental advocacy led to tension for the caregiver. On one hand, the stronger a caregiver’s parental advocacy skills, the better success they had at securing NDD-related supports. On the other hand, the more they had to advocate for services, the more frustration and stress they felt with structural inequities in having to advocate so hard for these services, which impacted negatively on quality of life.

In agreement with previous literature, caregivers also described the importance of provider-related facilitators (61). Families described how support from care coordinators in navigating the service structures, integrating caregivers’ needs in care planning, and the overall feeling of being supported improved their health, quality of life, family function, and satisfaction. However, caregivers emphasized that their family’s quality of life was integrated with their child’s health and that unmodifiable factors with their child’s disability reduced the degree of benefit they received from this intervention. They often cited that broader policies (ex.: COVID-19, school policies, admission criteria to services, etc.), were insufficient for their child’s health conditions and disabilities. As well, misunderstandings about these conditions from other members of the care teams (ex.: school personnel, other members of the clinical teams), and certain unmodifiable aspects of their children’s diagnosis were beyond the control of the care coordinators. Despite NDD-CC support, these factors impacted family quality of life, contributing to the lack of change or negative change observed in some participants.

#### The intersection of race and immigrant status for families of children with NDD-MC in clinical and care integration

The experiences of immigrant and Indigenous populations in navigating the CMC continuum of care are still not well understood and require further investigation. Through the addition of P10 and P12 in our study, these caregiver experiences contribute to the expansion of our knowledge with these populations. The P10 case study has implications for immigration-related policy-making by illustrating challenges with unemployment and unfamiliarity with community-based NDD-MC supports. Previous research showed that unemployment restricts access to NDD-MC resources in Canada (62) where most disability-related benefits and credits are delivered through the tax system (63). In most cases, families are required to pay upfront for services and apply for reimbursement which may be a barrier for low-income households. Immigration is a cornerstone of Canada’s Federal policy, where two-thirds of population growth is linked to international migration, with plans to add a further 500,000 immigrants annually until 2025 (64), and research shows that ASD is 36% higher in children of immigrants (62) adding urgency in understanding the challenges faced by this demographic. Furthermore, the immigration strategy aims to facilitate migration without overwhelming the health care system (24). Previous studies demonstrated the costs of inadequate access to NDD-MC supports. To improve policy outcomes, it is imperative that provincial and federal governments leverage families’ knowledge of health care, welfare, and community-based supports (62).

In 2007, the Canadian government passed Jordan’s Principle due to the impacts of payment disputes between different levels of government in funding health care for Indigenous populations (65). Despite Federal and provincial governments’ commitment to Indigenous populations, their perspectives and needs are underreported. This study contributes to addressing this research gap through the addition of P12 in our sample. This family had relocated from the city to a federal reserve where they struggled to access services and support. The care coordinator met them out of Calgary for several appointments and helped them access federal disability funding and community-based supports. The NDD-CC flexibility demonstrates possible ways in which CC can be adapted to accommodate changes to families’ circumstances. Further, it demonstrates that delivering culturally sensitive services should safeguard the mobility of Indigenous populations while preventing the loss of support from CC.

### Strengths, limitations and future research

Using a multilevel triangulation design provided several benefits to our study. First, a multimethod approach allowed us to use multiple research methods to identify a range of answers to a research question (66). Exploring this range is key in studies involving children with NDD-MC given the well-documented highly nuanced nature of their needs and individual circumstances. Previous CC studies have already demonstrated that a one-size fits all approach is counter effective in providing care for children with NDD-MC. Second, by integrating a multimethod approach we were able to conduct alternate levels of analysis (66). Analyzing individual-level, aggregate provincial-level, and group-level self-report datasets allowed us to develop a comprehensive understanding of the various system factors affecting the impact of CC interventions. Finally, a multimethod approach allowed us to offset the various counteracting limitations of individual methods, increasing the validity of our findings and simultaneously enhancing our understanding of the multifaceted nature (66) of CC interventions for children with NDD-MC. In addition, strengths of the study include the integration of the perspectives of immigrants and Indigenous populations. The understanding of the NDD-MC service trajectory of racial minority families with children with NDD-CC becomes increasingly important as Canada becomes more ethno-culturally diverse (62, 64).

The lack of a control group is a limitation of this study. As has been well documented in the literature (67), including a control group allows researchers to establish causality, measure the effectiveness of interventions, and reduce the risk of bias. Although our study shows promising findings regarding the impact of a CC intervention on families of children with NDD-MC and in reducing costs of care, a control group would have provided more ability to generalize the results to broader populations of families of children with NDD-MC. In lieu of a control group, participant data was compared to their outcomes prior to entering NDD-CC.

Future studies observing longer periods of health service utilization before and after CC would provide a more comprehensive picture as well as allow examination of whether the positive impacts of CC are sustained after families are discharged from the program. Another CC intervention showed that the number of unplanned hospital admissions and inpatient days was lower in the second year of program enrollment than in the first year (53, 55).

This study captured some of the nuances in the sociodemographic characteristics and NDD-MC diagnosis of families with NDD-MC, as evidenced in the recruitment strategy utilized for the qualitative interviews and the embedded case study component. Previous research has shown that different sociodemographic characteristics exert different levels of influence on the health and quality of life outcomes observed in families with children with NDD-MC (66, 68). However, assessing the extent of the influence of these factors is beyond the scope of this paper. Further research could consider a holistic analysis to evaluate the impact of counteracting factors on the impact of CC interventions.

Another limitation of this study is related to the COVID-19 pandemic. The inception and initial data collection of the study took place before the onset of the COVID-19 pandemic; however, data collection continued throughout the pandemic. In addition, due to the rolling recruitment approach, participants completed the surveys and interviews at the different times of the pandemic. Given that there were different waves to the pandemic (Figure 5), and each wave brought a unique set of challenges, it is likely that each family experienced different contexts based on the time of survey completion due to the different COVID-19 waves. In addition, due to the challenges brought by the pandemic we experienced loss to follow-up for the post CC intervention as families grappled with adjusting to the challenges imposed by the pandemic and the loss of services beyond CC to support their NDD-MC.

## Conclusion

This study expanded on the factors used to measure the outcomes of CC and adds to our understanding of how CC as an intervention impacts resource use, quality of life, and care integration of children with NDD-MC and their families. Given the heterogeneous nature of this population, evaluation studies that account for the variable and multi-level impacts of CC interventions is critical to inform practice, implementation, and policy of CC for children with NDD-MC. The NDD-CC project provides service navigation support, capacity-building for caregivers, and advocacy measures with broader care teams. The starting point of the NDD-CC journey varies for each family. Families often have additional needs which transcend the scope of CC. Our findings allude to the fact that the more integrated the families’ needs are with the areas that care coordination can directly impact, the more significant the improvements they experience.

Discussions regarding the impact of CC interventions often focus on assessing its influence in the broader medical, community, and education structures involved in supporting CMC. It is often the expectation that introducing CC interventions should address all the needs of a family with CMC. However, our findings have shown that the benefit that families are able to experience from CC interventions is often dependable on socioeconomic configuration, broader policies impacting supports and services, eligibility criteria to access services, attitudes, and perceptions of other members of the care teams, most of which are beyond the control of the NDD-CC project. Reducing policy disparities and policy reform is needed to further the impact of CC interventions. More consistent policies and availability of resources may lead to more sustainable CC interventions.

A successful care coordination intervention is going to be different for every family, given the heterogeneity of every circumstance. Our study informed us that although some families did not experience quantifiable improvements in their quality of life, resource use, and care coordination domains, they did feel supported, heard, and found an ally with their care coordinator which they stated made a successful impact. Disability policies in Canada are often criticized for treating disability as a transitory condition so perhaps the focus should shift to assessing the quality of support for families with medical complexity (what our cohort values) instead of trying to measure the ability of CC interventions to eradicate problems that cannot be eliminated.

## Data availability statement

The datasets presented in this article are not readily available because of patient confidentiality, participant privacy, limitations of Alberta’s Health Information Act, and the study’s ethics approval. Inquiries about the datasets should be directed to the corresponding author. Requests to access the datasets should be directed to zwicker1@ucalgary.ca.

## Ethics statement

The studies involving humans were approved by University of Calgary CHREB REB18-0743 and AHS Data Disclosure Agreement & Administrative Approval. The studies were conducted in accordance with the local legislation and institutional requirements. The participants provided their written informed consent to participate in this study.

## Author contributions

DM: Conceptualization, Data curation, Formal analysis, Investigation, Methodology, Software, Visualization, Writing – original draft, Writing – review & editing. GC: Conceptualization, Data curation, Formal analysis, Investigation, Methodology, Writing – original draft, Writing – review & editing. XJ: Conceptualization, Data curation, Formal analysis, Methodology, Software, Visualization, Writing – original draft, Writing – review & editing. BF: Data curation, Resources, Writing – review & editing, Validation. CR: Resources, Validation, Writing – review & editing. MY: Resources, Validation, Writing – review & editing. GL: Project administration, Resources, Validation, Writing – review & editing. ME: Validation, Writing – review & editing. TD: Writing – review & editing, Validation. SM: Methodology, Validation, Writing – review & editing. NG: Project administration, Resources, Writing – review & editing, Validation. BG: Conceptualization, Methodology, Writing – review & editing, Validation. JZ: Conceptualization, Data curation, Formal analysis, Funding acquisition, Investigation, Methodology, Project administration, Resources, Software, Supervision, Validation, Visualization, Writing – original draft, Writing – review & editing.
