# Supplementary material for: Measure what matters: considerations for outcome measurement of care coordination for children with neurodevelopmental disabilities and medical complexity
Source: Front Public Health. 2023 Nov 3;11:1280981. doi: 10.3389/fpubh.2023.1280981 (PMC10656699; doi:10.3389/fpubh.2023.1280981)
Supplement: Supplementary file 1 [file Data_Sheet_1.DOCX]

Measure what matters: considerations for outcome measurement of care coordination for children with neurodevelopmental disabilities and medical complexity

Dércia Materula^1^, Genevieve Currie^1,2^, Xiao Yang Jia^1^, Brittany Finlay^1^, Catherine Richard^3^, Meridith Yohemas^3^, Gina Lachuk^3^, Myka Estes^3^, Tammie Dewan^3^, Sarah MacEachern^3,4^, Nadine Gall,^3^ Ben Gibbard^3,5^, Jennifer D. Zwicker^1,6^*.

*** Correspondence:** Jennifer Zwicker: zwicker1@ucalgary.ca

**A case study on Winnie and her family (P12)**

**Introduction:** Winne is the aunt and primary caregiver of two grandchildren who have NDD-MC with complex behavioral issues. Grandmother and aunt are caregivers for six children. Indigenous family with a history of trauma and trust issues with providers. Struggling with finances regularly. Family moved to a rural residence just before care coordination commenced and children were going to new schools. No aide for one child in the classroom and speech therapy did not start with resource delays. This family was rated at Complexity 2 on the scale of complexity in the care coordination project. This family saw the care coordinator before the onset of the Covid-19 pandemic.

**Case description:**

**Broader economic, policy, social, and environmental influences.** Family overwhelmed with paperwork required in applications for community resources such as after school programs. Therefore, they were not accessing a number of resources in the community because of this. For quantitative data, no NDD-related services were accessed at baseline and 12-month. Pediatrician appointments were noted at baseline and 12-month surveys. No speech therapy and very little support for schoolwork during closure of schools with pandemic. After-school care was also stopped with pandemic restrictions. At baseline, grandmother noted two emergency department (ED) visits and no inpatient stays. Primary child being followed by care coordinator received care at 3 healthcare provider settings over 11 dates, totaling approximately $6243.11 in physician claims costs. At 12-month follow-up, this child had no ED visits and no inpatient stays. Child received care at 3 healthcare provider settings over 7 different dates, totaling approximately $1671.73 in physician claims costs.

At baseline, out of pocket (OOP) expenses were noted for school lunches (approximately $300); none were reported at 12-month. Caregiver reported no impacts of caregiving responsibilities on daily activities and employment at baseline and 12-month follow-up.

**Community environments, networks, and formal services.** Care coordination provided assistance with advocacy in school and medical appointments. One child transitioned to a new school after moving to live with aunt and grandmother. Better services and supports in school with coordinator attending meetings and explaining children’s needs and challenges to education and medical providers. Care coordinator accessed Big Brothers Big Sisters of Canada for children.

**Household: Function and Satisfaction.** Stress and negative impact on caregiver’s quality of life due to financial issues and lack of support. Winnie stated she was overwhelmed with trying to remember everything and coordinate the children’s care. Caregiver’s CarerQoL remained consistent at 75.9 and 75.3 at baseline and 12-month, respectively. The TS score decreased from 80 to 69, both scores in the normal range.

**Discussion:**

**Broader economic, policy, social, and environmental influences.** Winnie felt supported with access to resources in the community for family. Care coordinator provided support to complete the Boys and Girls club application for two children, after school programs, and provided access to bus tickets so child could get to school, food hampers, Christmas hampers, and completing income tax forms.

**Community environments, networks, and formal services.** Coordinator assisted with care planning and engaging with community-based providers. Winnie stated she felt coordinator supported her without judgement and particularly advocated with school providers when school absences occurred with child’s behavioral issues.

**Household: Function and Satisfaction**. This case study highlights assistance from the coordinator with addressing system navigation for medical, social care and educational needs. Caregiver stated she had less stress as the care coordinator integrated providers to meet and explain care goals to her. Also provided navigation to work within sectors and systems.

**A Case Study on Malik and his family (P10)**

**Introduction:** Parents were new to Canada. Child with NDD-MC had several (four) hospital admissions with mental health issues. Both parents were heavily involved in caregiving child. Malik the father, was unemployed for a period and struggling to care for child with medical and behavioral issues. This family was rated at Complexity 3 on the scale of complexity in the care coordination project. This family saw the care coordinator during Waves 1 and 2 of the Covid-19 pandemic.

**Case description:**

**Broader economic, policy, social, and environmental influences.** Family unaware of community-based supports to help with child’s complex care needs. This correlates with quantitative findings where caregiver reported no NDD-related services at baseline, and occupational therapist (OT) was accessed at 12-month. At baseline, had appointments with a pediatrician, psychiatrist, and psychologist. No appointments with specialists were noted by family at 12-month follow-up. At baseline, Malik noted no emergency dept. (ED) visits and three inpatient stays (72 days). Child received care at 4 healthcare provider settings over 93 dates, totaling approximately $2233.84 in physician claims costs. At 12-month follow-up, child had no ED visits and 1 inpatient stays (21 days). Child received care at 3 healthcare provider settings over 35 different dates, totaling approximately $503.98 in physician claims costs.

In addition, at baseline, Malik reported no missed daily activities and employment due to caregiving responsibilities. At 12 months, he reported dedicating 2-4 days per week to caregiving duties that affected employment and daily activities and his spouse gave up 70% of work hours to caregiving responsibilities, leading to reduced work hours for both. For quantitative findings, at baseline, no out of pocket expenses were noted and increased to over $7000 for special foods and supplements at 12-month follow-up.

**Community environments, networks, and formal services.** Lack of integration across systems and sectors with medical, educational and disability support. Family struggling to understand goals of care and manage care needs.

**Household: Function and Satisfaction.** Discussed several factors influencing quality of life (QoL) such as son’s health, finances, lack of privacy with in-home supports in the home, relationship strains with wife due to lack of privacy from care providers in the home and the need to care for son above all else. Focused however on child’s health issues as having the most impact on QoL and this has become their focus. Child experienced isolation and major transitions difficulties with school and home life from pandemic restrictions. “We were alone. We had to handle everything alone.” Malik felt mental health challenges worsened with lack of structure and routine from loss of school and community-based support. For quantitative findings, caregiver reported a reduction in the CarerQoL score from 86.5 to 75.8 (-10.7). The TS score increased from 105 to 125, going from the normal to the 98^th^ percentile category.

**Discussion:**

**Broader economic, policy, social, and environmental influences**. Care coordinator was able to equip family to access needed community-based resources to help with NDD. For example, care coordinator accessed in home supports for large portions of the day when the child was not in school to assist with feeding, dressing, indoor and outside home activities, and behaviors. Child required an out of home placement with medical and mental health challenges. Care coordinator was able to assist with advocating for placement with Family Supports for Children with Disabilities (FSCD).

**Community environments, networks, and formal services**. Care coordinator engaged with care planning with multiple providers to meet child's unique needs. For example, coordinator organized a case conference with family, psychiatrist, community pediatrician, in home supports, and mental health counsellor to coordinate care. Coordinator also attended meetings with FSCD. Also, individual meetings with school included the principal, teacher, and aides to determine care required. Coordinator also provided follow up meetings and reviewed minutes from team meetings with providers to make sure there were no gaps in follow up care.

**Household: Function and Satisfaction**. Family expressed that they were less stressed with support from care coordinator in working with child’s changeable medical, social and educational needs such as behavioral issues in school and providing in home supports for medical and mental health challenges. “Our care coordinator is our family supporter.”

**A Case Study on Cayley and her family (P17)**

**Introduction:** Cayley is a single mother living with her boyfriend and caring for child with multiple diagnoses and behavioral issues. Many care providers involved including medical specialists, dental provider and disability supports. Child also required assistance at schools with therapeutic, behavioral, and medical support. This family was rated as Complexity 3 on the scale of complexity in the care coordination project. This family saw the care coordinator during Waves 1 and 2 of the Covid-19 pandemic.

**Case description:**

**Broader economic, policy, social, and environmental influences.** Child was not coping in school as school setting and instruction not meeting child’s learning and behavioral needs. Also, family was not aware of resources that were available to assist them in the community with child’s NDD-MC needs.

Child accessed Occupational Therapy (OT), speech therapy (ST), psychologists at baseline and occupational therapist (OT) and speech therapist (ST) at 12-month. Child also had appointments with a pediatrician and neurologist at baseline and 12-month. At baseline, Cayley also noted no emergency dept. (ED) visits and 2 inpatient stays (3 days). Child received care at 2 healthcare providers setting over 15 dates, totaling approximately $1492.56 in physician claims costs. At 12-month follow-up, child had no ED visits and inpatient stays. Child received care at 2 healthcare provider settings over 5 different dates, totaling approximately $535.17 in physician claims costs. During the COVID lockdown measures, classes were delivered online but this was not workable for the child with NDD so he had no school. Medical appointments took place through Zoom platform. Respite was also delayed with pandemic restrictions. Cayley noted the stress on the family and on her son who required structure and consistency.

For quantitative findings at baseline, out of pocket, (OOP) expenses were noted for recreational activities, respite, medications, sleep app, and parking totaling approximately $2000. OOP expenses for respite, seizure tracking device, and sleep monitoring costing around $700 were reported at 12-month follow-up.

At baseline, Cayley noted caregiving responsibilities affected usual daily activities and/or employment 2-4 days per week, resulting in reduced work hours, using paid and unpaid leaves, and resignation; this was 1 day every 2 months for her spouse. No changes were noted at 12-month survey.

**Community environments, networks, and formal services.** Family were overwhelmed with new diagnosis of epilepsy and not feeling understood or supported by medical or educational team.

**Household: Function and Satisfaction.** Cayley discussed the impact of her son’s health as a major determinant of quality of life. Mentioned if her son was not happy and his health was not good this affected everyone. Mentioned her lack of having a normal job, which matters to her, and about her own mental health due to stress; discussed unpredictability of day to day life and how there are waves of difficulties. For quantitative findings, Cayley reported an increase in the CarerQoL score from 45.3 to 80.6 (+35.3).

**Discussion:**

**Broader economic, policy, social, and environmental influences.** Care coordinator assisted family to find and access resources and programs in the community to meet child’s care needs. For example, coordinator helped family to access a new school, connect resources offered by Family Supports for Children with Disabilities (FSCD), complete disability tax credit application, and access support from child development center.

**Community environments, networks, and formal services.** Coordinator helped with school and medical meetings to determine a plan of care. For example, care coordinator provided support to medical meetings with neurology and the new diagnosis of epilepsy, helped transition to a new school, and access to supports with Family Support for Children with Disabilities. Cayley was getting services in place for a behavioral aide but her son’s behavior improved. Was an advocate for Cayley and “someone in her corner” who understood her needs without judging her. Coordinator would bring up mom’s concerns at meetings if they were not addressed. Mom appreciated not getting the run around with no direct answer. “We had direct and informed answer once care coordination was involved.” Cayley feels medical team and school were more amenable to answering questions and providing access to services if coordinator was present. Cayley said that before care coordination she “felt like I was being trampled…” when discussing how she has had to fight for care support. Cayley stated she felt more confident now herself at meetings to ask the hard questions.

**Household: Function and Satisfaction.** Mom expressed that care coordination helped the family deal with the constant crises from her child’s medical and emotional health.

**A Case Study on Sarah and family (P19)**

**Introduction:**

Family had care of child with NDD - MC as well as father had chronic medical issues. Child was experiencing difficulties with schooling and bullying at school. Sarah expressed there was a lack of support from school principal regarding her child’s needs. Sarah eventually pulled her child out of the school and chose a new school with the support of care coordinator. Coordinator also assisted with accessing financial resources. This family was rated as Complexity 2 on the scale of complexity in the care coordination project. The family saw the coordinator during Wave 1 of the Covid-19 pandemic.

**Case description:**

**Broader economic, policy, social, and environmental influences.** Family had hours for respite, childcare for appointments and therapy costs but had to wait for pandemic restrictions to lift to be able to access these services. This correlates with quantitative findings where no NDD-related services were accessed at baseline and 12-month.

Child had appointments with a pediatrician and family doctor at baseline. Appointments with the pediatrician, family doctor, and neurologist were reported at 12-month. At baseline, Sarah noted no ED visits and inpatient stays for her child. Child received care at 2 healthcare providers setting over 5 dates, totaling approximately $417.23 in physician claims costs. At 12-month follow-up, child had no ED visits and inpatient stays. Child received care at 5 healthcare provider settings over 13 different dates, totaling approximately $1435.55 in physician claims costs.

Sarah cited no impacts on daily activities or employment at baseline due to caregiving responsibilities and 7 days each for her and her spouse at 12-month. In addition, for quantitative findings at baseline, out of pocket, (OOP) expenses totaled approximately $600 for special materials and education assessment were noted. Parking expenses of $30 were reported at 12-month.

**Community environments, networks, and formal services.** Family was experiencing lack of understanding of medical needs of child and lack of communication with neurology clinic. Child was also experiencing bullying at school with little support to change situation with school. Also family was new in the process of getting a contract with Family Supports for Children with Disabilities (FSCD).

**Household: Function and Satisfaction.** Sarah described that her quality of life (QOL)suffered with coordinating numerous medical appointments for her son. This lessened with COVID-19 since the appointments were not happening with restrictions. She also discussed the impact of her son not being happy and bullying at school as a major determinant of Q of L as her son’s mood and outlook affects everyone. Sarah also shared she was seeing a therapist for her own mental health due to stress and she needed this on a regular basis. For quantitative findings, caregiver reported an increase in the CarerQoL score from 84.6 to 92.4 (+7.8). The TS score decreased from 118 to 84, going from the clinically significant category to the normal range.

**Discussion:**

**Broader economic, policy, social, and environmental influences.** Care coordinator was not able to help family access resources to assist with NDD-MC such as respite, therapy and childcare for appointments as these supports were no longer available during the early phases of the Covid-19 pandemic.

**Community environments, networks, and formal services.** Coordinator orchestrated many meetings in care planning with engaging school providers, administrators, FSCD worker and neurology medical team to address issues experienced by the child and family. For example, care coordinator intervened with principal, assistant principal, school psychologist, and school liaison officer to try and make changes with bullying experienced at the school. Coordinator brought forward Sarah’s concerns at meetings if they were not addressed. Care coordinator also debriefed with Sarah following the meetings. Sarah shared care coordinator helped the team to be answerable to outstanding issues. Coordinator has not been able to help with utilizing COVID resources, as Sarah could not access these resources until after COVID. “She was like my shield.” “She made me feel that I had a suit of armor on before I went into those [school] meetings.”

**Household: Function and Satisfaction*.*** Care coordinator assisted with changing care needs with child. For example, CC worked with the medical team and many school providers to address the issues of the child being bullied. She supported the mother during school meetings, which Sarah commented on as making a difference in reducing her stress and providing a shield for her in the meetings.
